# PRMT1 methylation of WTAP promotes multiple myeloma tumorigenesis by activating oxidative phosphorylation via m6A modification of NDUFS6

**DOI:** 10.1038/s41419-023-06036-z

**Published:** 2023-08-09

**Authors:** Yachun Jia, Xiao Yu, Rui Liu, Luyi Shi, Hua Jin, Dan Yang, Xiaofeng Zhang, Ying Shen, Yuandong Feng, Peihua Zhang, Yi Yang, Linlin Zhang, Pengyu Zhang, Zongfang Li, Aili He, Guangyao Kong

**Affiliations:** 1 National and Local Joint Engineering Research Center of Biodiagnosis and Biotherapy, The Second Affiliated Hospital of Xi’an Jiaotong University, Xi’an, Shaanxi P. R. China; 2 Department of Hematology, The Second Affiliated Hospital of Xi’an Jiaotong University, Xi’an, Shaanxi P. R. China; 3 Precision Medical Institute, The Second Affiliated Hospital of Xi’an Jiaotong University, Xi’an, Shaanxi P. R. China; 4 Key Laboratory of Environment and Genes Related to Diseases, Xi’an Jiaotong University, Xi’an, Shaanxi P. R. China

**Keywords:** Myeloma, Methylation

## Abstract

Epigenetic modifications play important roles during the pathogenesis of multiple myeloma (MM). Herein, we found that protein arginine methyltransferase 1 (PRMT1) was highly expressed in MM patients, which was positively correlated with MM stages. High *PRMT1* expression was correlated with adverse prognosis in MM patients. We further showed that silencing *PRMT1* inhibited MM proliferation and tumorigenesis in vitro and in vivo. Mechanistically, we revealed that the knockdown of *PRMT1* reduced the oxidative phosphorylation (OXPHOS) of MM cells through NDUFS6 downregulation. Meanwhile, we identified that WTAP, a key component of the m^6^A methyltransferase complex, was methylated by PRMT1, and NDUFS6 was identified as a bona fide m^6^A target of WTAP. Finally, we found that the combination of PRMT1 inhibitor and bortezomib synergistically inhibited MM progression. Collectively, our results demonstrate that PRMT1 plays a crucial role during MM tumorigenesis and suggeste that PRMT1 could be a potential therapeutic target in MM.

## Introduction

Multiple myeloma (MM) is a fatal hematologic tumor characterized by abnormal proliferation of monoclonal plasma cells in the bone marrow [1]. Current evidence suggests that the average age of MM diagnosis is around 70 years [2]. While several effective treatment strategies for this patient population have emerged in recent decades, including proteasome inhibitors, immunomodulatory drugs, monoclonal antibodies, chimeric antigen receptor-engineered T cells, and autologous stem cell transplantation [3–6], MM remains incurable due to the onset of drug resistance and relapse [7]. It is now understood that various factors are involved in MM progression, mainly including genetic abnormalities [8], changes in the bone marrow microenvironment [9], and epigenetic alterations [10]. Nonetheless, the etiology of MM remains unclear, highlighting the need to understand the molecular mechanisms of MM patients and identify novel therapeutic targets.

Protein arginine methyltransferases (PRMTs) are enzymes that catalyze the methylation of arginine residues on histones and non-histone proteins and can be classified into three major types: type I (PRMT1-4, PRMT6, PRMT8), type II (PRMT5, PRMT9), or type III (PRMT7) [11]. PRMTs participate in various biological processes, including signal transduction, epigenetic regulation, and DNA repair [12]. Among the PRMT family members, PRMT1 is the predominant type I enzyme responsible for the monomethylation and 85% of asymmetric dimethylation of arginine residues in mammalian cells [13]. Over the years, PRMT1 has been associated with the progression of various diseases, especially cancers [14]. For instance, PRMT1 could promote lung cancer growth and breast cancer metastasis [15, 16]. Besides, PRMT1 could enhance the oncogenic arginine methylation of NONO in colorectal cancer via asymmetrically demethylating R251 of NONO [17]. However, the function of PRMT1 in MM progression remains unclear, warranting further research.

In this study, we provide hitherto undocumented evidence of elevated *PRMT1* expression in MM and its correlation with adverse outcomes in this patient population. Further exploration demonstrated that loss of *PRMT1* inhibited MM proliferation in vitro and in vivo and caused oxidative phosphorylation (OXPHOS) dysfunction. Mechanistically, we found that PRMT1 affected the function of mitochondria by regulating NDUFS6 expression via an m^6^A-WTAP-dependent pathway. Finally, we revealed the synergistic cytotoxic effect of PRMT1 inhibitor and bortezomib (BTZ) in MM cell lines and patient primary cells. Overall, our findings provide a potential therapeutic approach for MM by targeting PRMT1.

## Methods

### Patient samples and databases used

In this study, bone marrow aspirates were collected from 23 normal donors and 31 newly diagnosed multiple myeloma (NDMM) patients. The study protocol was approved by the Ethics Committee of the second affiliated hospital of Xi’an Jiaotong University, Shaanxi, China (#2022128). Written informed consent was obtained from all patients. Among the MM patients, there were 16 males and 15 females aged from 37 to 82 years. (Supplementary Table 1). Primary MM CD138^+^ cells were obtained using CD138 beads (MicroBeads, human, Miltenyi Biotec, #130-051-301, Germany) for purification. The diagnosis, stage, and risk status of MM were made in accordance with the National Comprehensive Cancer Network (NCCN) (2020 version 4) and mSMART 3.0. Besides, MM mRNA expression and clinical features were downloaded from The Cancer Genome Atlas (TCGA, UCSC Xena, https://xenabrowser.net/) and Gene Expression Omnibus (GEO, https://www.ncbi.nlm.nih.gov/geo/) databases.

### Bioinformatics analysis

MM patients were divided into two groups upon the optimal cutoff of the gene expression with the "Survminer" package (version 0.4.9). Kaplan-Meier survival analysis with log-rank test was used to evaluate the differences in survival rate between the two groups using the "SURVIVAL" package (version 3.2-7) [18]. Univariate and multivariate Cox regression analyses were conducted to determine the significance of various factors for MM prognosis. Variables with *p* < 0.05 during multivariate Cox regression analysis were regarded as independent prognostic factors. Then, we used the "rms" package (version 6.2-0) to construct a nomogram. Finally, we assessed the predictive value of the nomogram by receiver operating characteristic (ROC) curve analysis and generating calibration curves.

### Reagents

Bortezomib (HY-10227, purity ≥ 98.0%) was purchased from MCE. C7280948 (S6737, purity ≥ 98.0%) was from Selleck Chemicals. These drugs were dissolved in Dimethyl sulfoxide (DMSO), aliquoted, and stored at −20 °C.

### Establishment of overexpressed (OE) and knockdown (KD) MM cell lines

Transient transfection was performed with siRNA targeting negative control (NC), *PRMT1*, *NDUFS6*, *METTL3*, *METTL14*, *WTAP*, and *YTHDF2* at a final concentration of 100 nM using Lipofectamine RNAiMAX according to the manufacturer’s instructions (Thermo Fisher Scientific, China). All siRNAs were purchased from Ribobio (Guangzhou, China). The siRNA sequences used are presented in Supplementary Table 2. MM cells were transfected with overexpression plasmids of PRMT1 and WTAP using Lipofectamine 3000 according to the manufacturer’s instructions (Thermo Fisher Scientific, China). Following transfection for 72 h, cells were collected to examine knockdown and overexpression efficiency by qRT-PCR and western blotting.

### Cell proliferation and cytotoxicity assays

Cell proliferation was detected by Cell Counting Kit-8 (CCK8, Beyotime, Shanghai, China) and EdU-staining assays. MM cell lines were seeded into 96-well plates (5×10^3^ cells /well) and transfected with siRNA or plasmids for 5 consecutive days. Cell viability was examined using the absorbance at 450 nm by a microplate reader, and 10 μl of CCK-8 solution was added to each well 2 hours in advance.

The EdU staining assay was performed by BeyoClickTM EdU cell proliferation Kit with Alexa Fluor 647 (Beyotime, Shanghai, China). 1×10^5^ MM cells transfected with siRNA or plasmids were cultured in 24-well plates. After 72 h, cells were maintained with 10 μM EdU at 37 °C for 2 h. Next, MM cells were fixed, permeabilized, and incubated with a click reaction solution in accordance with the manufacturer’s protocols. Finally, the APC-positive cells were identified by flow cytometry.

Cell cytotoxicity was detected by CCK8 assays. MM cell lines (5×10^3^ cells /well) or primary MM CD138^+^ cells (1×10^4^ cells /well) were seeded into 96-well plates treated with single drugs or combinations for 48 h. The half-maximal inhibitory concentration (IC_50_) and Combination Index (CI) were calculated using Compusyn software. A CI value < 1 indicated synergism.

### Cell apoptosis assays

The cell apoptosis rate was detected by Annexin V-PE/RedNucleus II assay from Bioscience (Shanghai, China). MM cell lines were transfected with siRNA or plasmids and cultured in 24-well plates (1 × 10^5^/well). After 72 h, 5 μL Annexin V-PE and 5 μL RedNucleus II were added into cells for 20 minutes at room temperature in the dark. Then flow cytometry was used to assess MM cell apoptosis. The results were analyzed with FlowJo software.

### Measurement of reactive oxygen species (ROS)

MM cell lines were transfected with siRNA or plasmids and cultured in 24-well plates (1×10^5^/well). After 72 h, cells were maintained with 10 μM DCFH-DA (Bioscience, Shanghai, China) at 37 °C for 30 min in the dark and washed with PBS. Then flow cytometry was used to assess FITC-positive MM cells. The results were analyzed with FlowJo software.

### Measurement of oxygen consumption (OCR)

OCR was measured using a Seahorse XF96 Extracellular Flux Analyzer (Agilent, Chicopee, USA). Briefly, MM cells (4 ×10^4^/well) transfected with siRNA or plasmids for 72 h were seeded in a Seahorse culture plate precoated with Cell-Tak. OCR was finally measured by sequential addition of oligomycin (1.5 μM), FCCP (1 μM), and Rotenone/antimycin A (0.5 μM). The results were analyzed with online Seahorse Analytics.

### Animal experiments

Female BALB/c nude mice (4-5-week-old) were purchased from (Beijing, China) and fed in specific pathogen-free facilities with 12 h light/12 h dark. Following *PRMT1* knockout, MM.1 S cells (5 ×10^6^) were resuspended in 200 μL PBS and subcutaneously injected into mice. To assess the synergistic cytotoxic effect of inhibiting PRMT1 and BTZ, the mice were randomly divided into different treatment groups. The control group consisted of mice injected with sg-NC MM cells and received no treatment. The BTZ group included mice injected with sg-NC MM cells and treated with BTZ alone. The combination group included mice injected with sg-PRMT1 MM cells and treated with BTZ. The BTZ treatment involved administering BTZ at a dose of 1 mg/kg, which was dissolved in a solution containing 2% DMSO, 30% PEG 300, and ddH_2_O. The BTZ treatment was administered twice a week through intraperitoneal injection for a duration of 24 days.Tumor size was measured every day. Tumor volume was calculated as follows: length × width^2^ × 0.5. The mice were sacrificed when the tumor diameter reached 15 mm. All animal experiments were approved by the Committee on Animal Research and Ethics of the second affiliated hospital of Xi’an Jiaotong University (#2022128).

### Statistical analysis

Three dependent biological replicates were performed in each experiment, and values were presented as mean ± SD. We used unpaired Student’s t-test and Mann-Whitney U test to determine the significance between the two groups. For three or more groups, we used One-way ANOVA (for parametric data) and Kruskal-Wallis (for non-parametric data) tests to compare significance. The correlation between *PRMT1* with OXPHOS-related genes was assessed by Pearson correlation analysis. ROC curve was used to calculate the diagnosis value of *PRMT1* and the prognostic value of the nomogram model in MM. The Kaplan-Meier method with a two-sided log-rank test was used to assess the overall survival rate and poor progression-free survival rate of MM patients. SPSS 21 software (SPSS, Chicago, USA) and GraphPad Prism 8 were used for statistical analysis. *A p-*value less than 0.05 was statistically significant.

## Results

### High *PRMT1* expression was related to adverse outcomes in MM patients

**Fig. 1:**
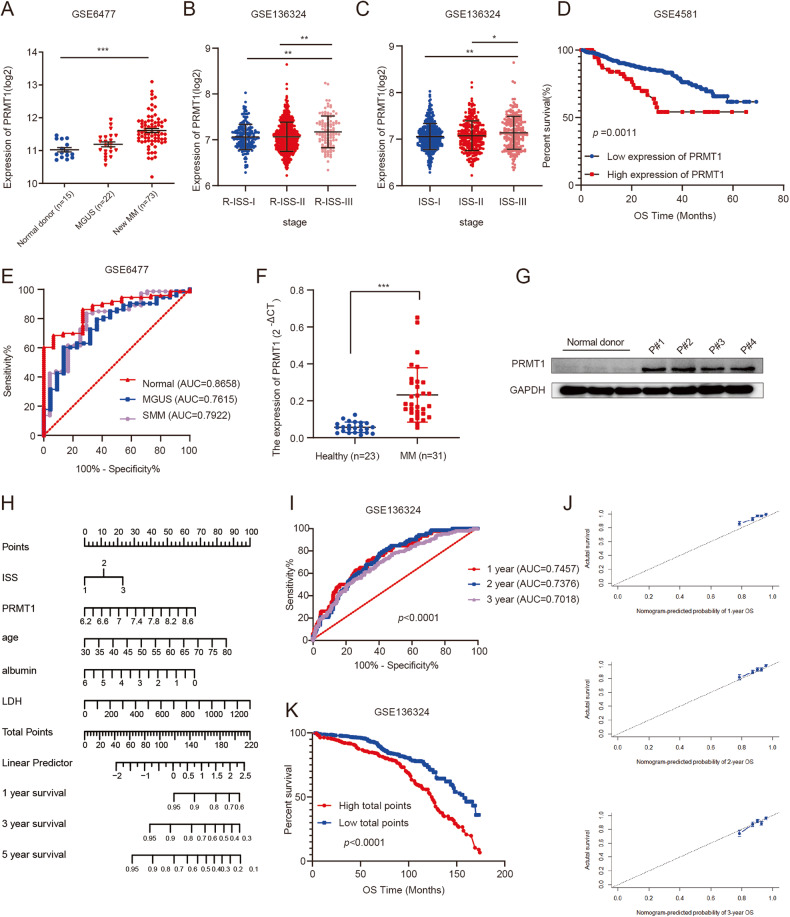
High PRMT1 expression was related to adverse outcomes in MM patients. **A-C**. *PRMT1* expression in normal donors and MM patients (**A**), MM patients with different R-ISS stages (**B**), or MM patients with different ISS stages **(C**) in GSE6477 and GSE136324, respectively. **D** Kaplan-Meier survival analysis of overall survival rate in GSE4581. **E** ROC curve for MM with different myeloma stages. PRMT1 mRNA expression (**F**) and protein expression (**G**) in primary bone marrow mononuclear cells from NDMM and normal donors. **H** Nomogram predicting 1-, 3-, and 5-year OS for MM patients based on *PRMT1* and other prognostic parameters. **I** ROC curve for the nomogram prognosis system. **J** Calibration plot of the nomogram for 1-year, 2-year, and 3-year OS. (**K**) Kaplan-Meier survival analysis for total points of Nomogram. Data represent the mean ± SD.* *p* < 0.05, ** *p* < 0.01, *** *p* < 0.001.

To investigate the role of PRMT1 in MM, public datasets were queried to explore the potential clinical value of *PRMT1* in MM and different MM stages. Analysis of dataset GSE6477 revealed that *PRMT1* expression was significantly increased in 73 NDMM patients compared with 15 normal donors (*p* < 0.0001, Fig. 1A). Besides, analysis of dataset GSE13591 revealed significant upregulation of *PRMT1* expression in MM (n = 133, *p* = 0.016), with even more pronounced elevation in plasma cell leukemia (PCL) (n = 9, *p* < 0.0001, Supplementary Fig. 1A). Similar findings were observed in dataset GSE2113 (*p* = 0.001, *p* = 0.004, Supplementary Fig. 1B). Furthermore, we found MM patients with higher R-ISS stage in TCGA cohort (*p* = 0.009, *p* = 0.02, Supplementary Fig. 1C) and GSE136324 (*p* = 0.0023, *p* = 0.0014, Fig. 1B) and ISS stage in GSE136324 (*p* = 0.0022, *p* = 0.0368, Fig. 1C) exhibited higher levels of *PRMT1* expression. Next, we analyzed the prognostic value of *PRMT1* in MM. We found higher *PRMT1* expression correlated with adverse overall survival (OS) rate among MM patients from dataset GSE4581 (*p* = 0.044, Fig. 1D), GSE24080(*p* = 0.027, Supplementary Fig. 1D), and TCGA (*p* < 0.0001, Supplementary Fig. 1E). Meanwhile, higher *PRMT1* expression was correlated with poorer progression-free survival (PFS) in GSE136324 (*p* < 0.0001, Supplementary Fig. 1F). ROC curve analysis (Fig. 1E) suggested that *PRMT1* expression could differentiate MM from normal donors (AUC = 0.8658, 95% confidence interval (CI): 0.7833 to 0.9482, *p* < 0.0001), monoclonal gammopathy of undetermined significance (MGUS) (AUC = 0.7615, 95%CI: 0.6515 to 0.8715, *p* = 0.002) and smoldering multiple myeloma (SMM) (AUC = 0.7922, 95%CI: 0.6878 to 0.8967, *p* < 0.0001).

In addition, we compared PRMT1 expression in primary bone marrow mononuclear cells from NDMM and normal donors. *PRMT1* mRNA expression (*p* < 0.0001, Fig. 1F) was increased in NDMM patients compared to controls. Consistently, NDMM patients exhibited higher PRMT1 protein expression (Fig. 1G) than normal donors.

### *PRMT1* expression was an independent prognostic factor for MM survival

Based on the above findings, we explored the correlation between *PRMT1* expression and patient characteristics in the GSE136324 dataset (n = 844 MM patients) (Table 1). We observed that *PRMT1* expression was correlated with MM patient age (*p* = 0.042), beta-2 microglobulin (B2M) levels (*p* < 0.0001), and the ISS stage (*p* = 0.005). In addition, GSE136324 was used for univariate and multivariate Cox regression analysis. The results showed that age (hazard ratio (HR), 1.046; 95%CI, 1.032-1.061; *p* < 0.0001), lactate dehydrogenase (LDH) (HR, 1.002; 95% CI, 1.001-1.003; *p* = 0.003), albumin (ALB) (HR, 0.746; 95% CI, 0.642-0.866; *p* < 0.0001), ISS stage (HR, 1.355; 95% CI, 1.113-1.650; *p* = 0.002), and *PRMT1* expression (HR, 1.966; 95% CI, 1.400-2.759; *p* < 0.0001) were independent prognostic factors for survival of MM patients (Supplementary Table 3). To facilitate clinical implementation, a nomogram model was constructed incorporating the above independent prognostic factors (Fig. 1H). The nomogram allowed the calculation of total points based on the values assigned to each factor, providing an estimate of an individual patient’s survival probability. ROC and calibration curves were used to evaluate the accuracy and efficacy of the prognostic model. The AUC value of the total points in nomogram for OS at 1, 2, and 3 years was 0.7457 (95%CI: 0.6776 to 0.8138, *p* < 0.0001), 0.7376 (95%CI: 0.6853 to 0.7898, *p* < 0.0001), and 0.7018 (95%CI: 0.6503 to 0.7532, *p* < 0.0001), respectively (Fig. 1I). Calibration curves are typically used to assess the agreement between predicted probabilities and observed event rates or frequencies in the real world, with the 45° line representing the best prediction scenario. In the present study, the calibration curves revealed good agreement between predicted and actual outcomes (Fig. 1J). Moreover, we analyzed the prognostic value of total points in MM. We divided MM patients into high and low total points groups based on the optimal cutoff of the total points. The Kaplan-Meier survival analysis showed MM patients with higher total points showed significantly adverse OS (*p* < 0.0001, Fig. 1K). Taken together, we identified that increased *PRMT1* was associated with poor prognosis in MM and could be used as a novel biomarker. *PRMT1* expression was the independent prognostic factor for MM survival. Our nomogram based on *PRMT1* expression exhibited good performance in predicting OS in MM patients.

**Table 1:** Patients’ characteristics in the GSE136324 dataset of 844 MM patients according to PRMT1 expression levels.

characteristics		PRMT1 low	PRMT1 high	*p*
Age, mean(range)		58.51 (32.4–75.2)	56.56 (35.8–73.3)	**0.042**
Gender (%)	female	267 (31.64%)	51 (6.04%)	0.065
B2M(mean(range))	male	465 (55.09%) 4.6 (0.45–49.1)	61 (7.23%) 5.7 (1.1–28.6)	**<0.0001**
LDH (mean(range))		158.7 (73–666)	187.4 (68–1278)	0.444
ALB (mean(range))		3.87 (0.3–5.8)	3.85 (1.9–5.8)	0.434
ISS (%)	I	171 (20.3%)	17 (2.0%)	**0.005**
	II	469 (55.6%)	71 (8.4%)	
	III	92 (10.90%)	24 (2.8%)	

Bold values indicates statistically significant *p* values less than 0.05.

### Downregulation of *PRMT1* inhibited MM progression in vitro and in vivo

**Fig. 2:**
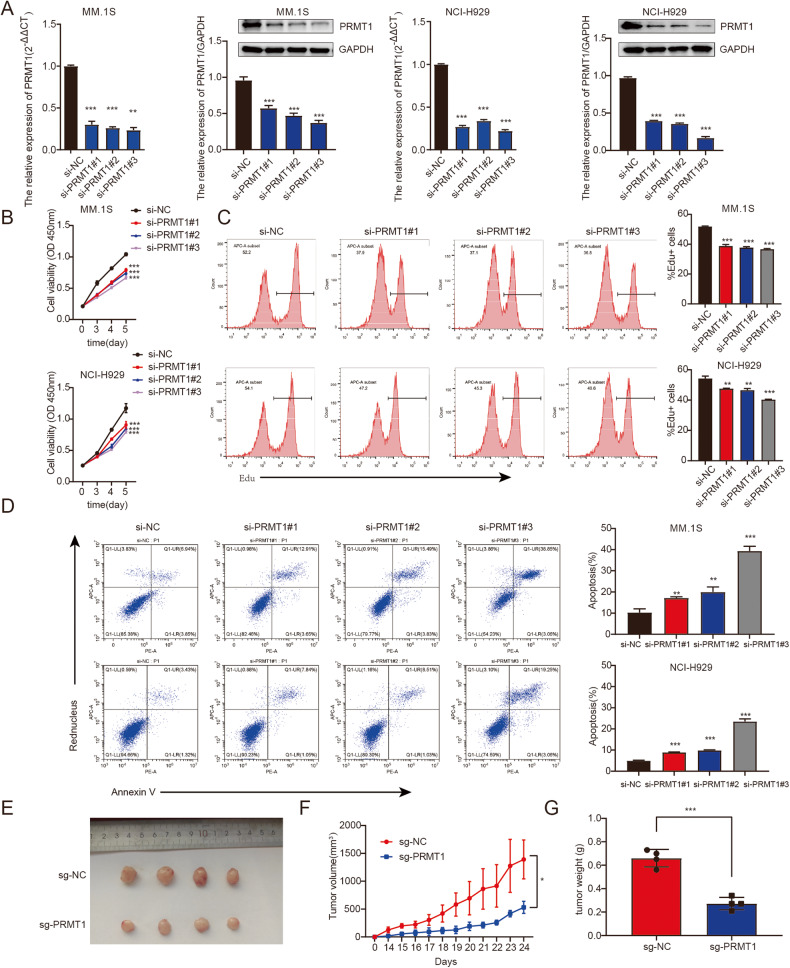
Downregulation of PRMT1 inhibited MM progression in vitro and in vivo. **A** The mRNA and protein of PRMT1 was detected by qRT-PCR and western blotting in MM.1 S and NCI-H929 cells transfected with si-NC or si-*PRMT1*. Total RNA was extracted after 48 h and protein was extracted after 72 h following the transfection. **B**, **C** Proliferation of MM cells transfected with si-NC or si-*PRMT1*, as determined by CCK8 (0, 3,4, and 5 days) (**B**) and EDU-staining (after 72 h following the transfection) (**C**). **D** Apoptotic analysis of MM cells by flow cytometry after 72 h following the transfection. **E** Photographs of tumors. **F** Quantification of the tumor volumes in nude mice after implantation of MM.1 S sg-NC and sg-PRMT1 (mean tumor volume±S.D., 4 mice per group). **G** Quantification of tumor weights from MM-xenograft mice on day 24. Data represent the mean ± SD. Experiments were performed in triplicate. * *p* < 0.05, ** *p* < 0.01, *** *p* < 0.001.

Our findings indicated that PRMT1 may be involved in the development of MM. To confirm the function of PRMT1, we first examined its expression in MM cell lines. We found PRMT1 was expressed in MM cell lines at both the mRNA and protein levels (Supplementary Fig. 2A-B). Next, gain- and loss-of-function studies were performed to explore the role of PRMT1 in MM. The knockdown and overexpression efficiency were examined by qRT-PCR and western blotting (Fig. 2A, Supplementary Fig. 2C-D). The results showed that silencing *PRMT1* could significantly inhibit MM cell growth and proliferation (Fig. 2B-C) but increased cell apoptosis (Fig. 2D) in MM.1 S and NCI-H929 cells. Consistently, PRMT1 overexpression enhanced MM cell growth and proliferation in NCI-H929 and RPMI-8226 cells (Supplementary Fig. 2E-F) and decreased cell apoptosis (Supplementary Fig. 2G) in MM.1 S and RPMI-8226 cells. To further confirm our findings, we knocked out *PRMT1* in MM.1 S using CRISPR-Cas9, and the expression of PRMT1 was verified by western blotting. We found PRMT1 expression was suppressed in the sg-PRMT1-3-2 group (Supplementary Fig. 3A). Then, we subcutaneously injected 5 million MM.1 S cells transfected with sg-NC or sg-PRMT1-3-2 into nude mice. The results showed that *PRMT1* knockout significantly restrained tumor volume and reduced tumor weight (Fig. 2E-G). Hematoxylin-eosin (H-E) staining of tumor tissues showed an increase in necrotic areas in the *PRMT1* knockout group (Supplementary Fig. 3B). In addition, the immunohistochemical analysis indicated a decrease in the number of Ki-67 positive cells due to *PRMT1* knockout (Supplementary Fig. 3B). Taken together, these finding indicated that loss of PRMT1 could delay MM progression in vitro and in vivo.

### Downregulation of *PRMT1* inhibited oxidative phosphorylation of MM cells

**Fig. 3:**
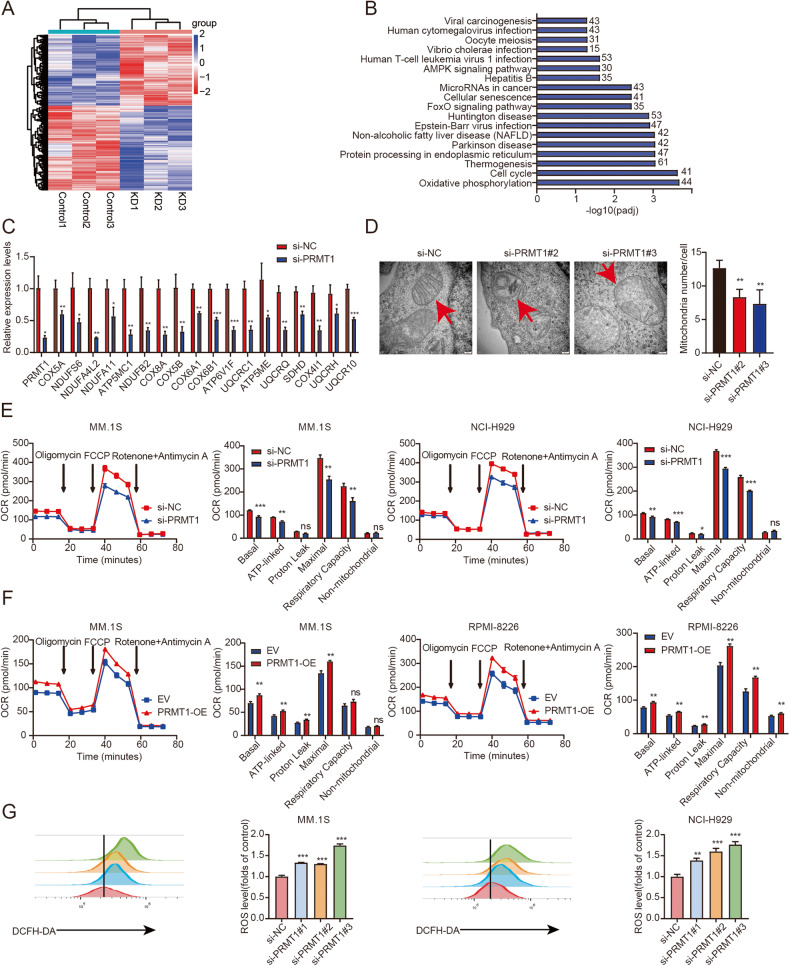
Downregulation of PRMT1 inhibited oxidative phosphorylation of MM cells. **A** Heatmap of RNA-seq analysis of MM cells upon NC and *PRMT1* KD after 72 h following the transfection. **B** KEGG analysis of the genes with significantly changed in *PRMT1* KD cells. **C** The expression of OXPHOS genes was verified by qRT-PCR. **D** Different in mitochondrial morphology in MM.1 S NC and *PRMT1* KD cells after 72 h following the transfection by TEM. The quantification represents the number of mitochondrial in each cell. Scale bar, 200 nm (×50000). **E** Measurement of OCR in MM.1 S and NCI-H929 cells transfected with si-NC and si-*PRMT1* after 72 h. **F** Measurement of OCR in MM.1 S and RPMI-8226 cells transfected with EV and PRMT1-OE after 72 h. **G** Reactive oxygen species (ROS) levels were assessed by flow cytometry transfected with si-NC and si-*PRMT1* after 72 h. Data represent the mean ± SD. Experiments were performed in triplicate. * *p* < 0.05, ** *p* < 0.01, *** *p* < 0.001. n.s. not significant.

To further explore the underlying mechanism of PRMT1 in MM, RNA sequencing (RNA-seq) was performed in MM.1 S cells transfected with si-NC or si-*PRMT1*. As illustrated in Fig. 3A, 2731 genes were differentially expressed after *PRMT1* knockdown (*PRMT1* KD), including 1244 downregulated and 1487 upregulated genes. KEGG analysis showed several pathways were enriched, including OXPHOS, cell cycle, Foxo signaling pathway, and AMPK signaling pathway (Fig. 3B). Next, qRT-PCR was performed and confirmed that most OXPHOS-related genes were significantly downregulated in *PRMT1* KD cells (Fig. 3C). Subsequently, the OXPHOS capacity was further assessed by examining mitochondrial morphology and activity [19]. The transmission electron microscopy (TEM) results indicated mitochondrial abnormalities in *PRMT1* KD cells, including swelling, decreased electron density, absence of cristae, vacuolation, and reduced mitochondrial numbers (Fig. 3D). Then, we measured the oxygen consumption rate (OCR) by a Seahorse XF96 analyzer. As expected, *PRMT1* KD decreased OCR parameters, including the basal, ATP-linked, maximal, and spare respiratory capacity in both MM.1 S and NCI-H929 cells (Fig. 3E). Consistently, PRMT1 overexpression increased the OCR in both MM.1 S and RPMI-8226 cells (Fig. 3F). Besides, *PRMT1* KD significantly increased ROS levels (Fig. 3G), while PRMT1 overexpression decreased ROS levels in both MM.1 S and NCI-H929 cells (Supplementary Fig. 4A). Accordingly, we focused on OXPHOS-related genes. We found that the expression of *COX5A*, *COX5B*, *NDUFS6*, *COX8A*, and *ATP6V1F* was higher in MM patients than in normal donors based on the GSE13591 dataset (Supplementary Fig. 4B). In addition, we analyzed the prognostic value of these 5 genes. Similarly, a significant correlation was observed between the higher expression of *COX5A*, *COX5B*, *NDUFS6*, *COX8A*, and *ATP6V1F* in MM and poor OS in TCGA cohort (Supplementary Fig. 4C). We further validated the significant positive correlation between *PRMT1* and the 5 genes in TCGA cohort (Supplementary Fig. 4D). Taken together, these results demonstrated PRMT1 induced OXPHOS activation by influencing genes related to OXPHOS in MM.

### NDUFS6 was the potential downstream effector of PRMT1 in MM

**Fig. 4:**
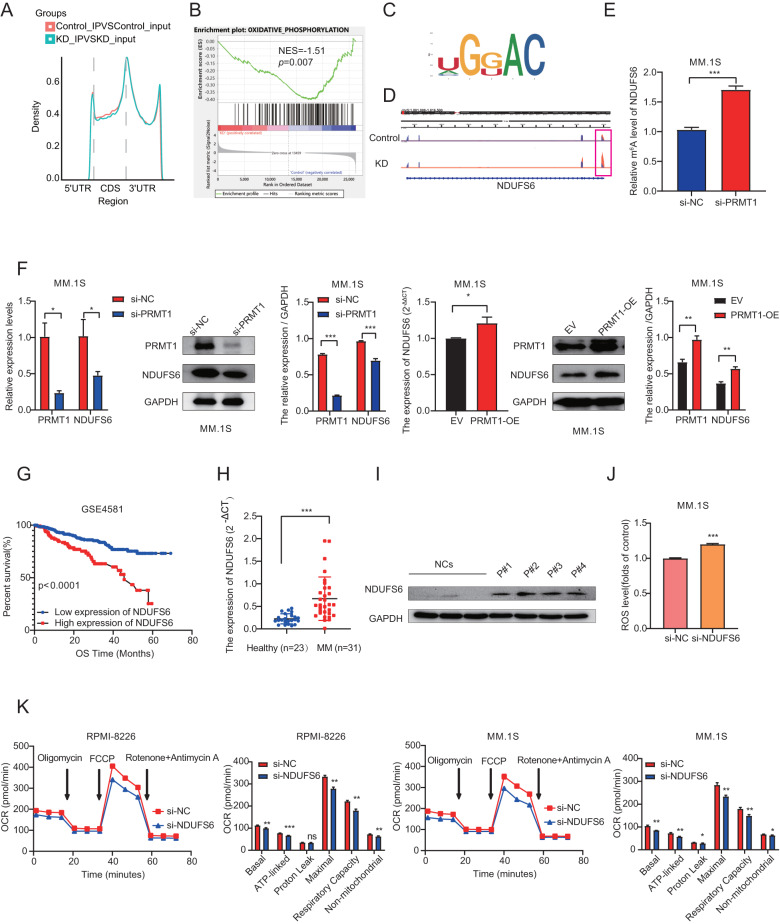
NDUFS6 was the potential downstream effector of PRMT1 in MM. **A** The density distribution of total m^6^A peaks in the NC and *PRMT1* KD cells. **B** GSEA analysis of differentially expressed genes in *PRMT1* KD cells based on m^6^A -seq. **C** Predominant consensus motif GGAC was detected in both NC and *PRMT1* KD cells. **D** Integrative Genomics Viewer (IGV) tracks display m^6^A abundance in NDUFS6 transcripts in NC and *PRMT1* KD cells. (E) MeRIP-qPCR analysis m^6^A enrichment of NDUFS6 transcripts in MM.1 S cells with or without si-*PRMT1* 72 h after transfection. **F** qRT-PCR and western blotting analysis NDUFS6 mRNA and protein level in MM.1 S cells transfected with si-NC or si-*PRMT1*, or EV and PRMT1-OE cells after 72 h. **G** Kaplan-Meier survival analysis of OS in GSE4581. NDUFS6 mRNA expression **H** and protein expression (**I**) in primary bone marrow mononuclear cells from NDMM and normal donors. **J** ROS level was assessed by flow cytometry in MM.1 S NC and *NDUFS6*-KD cells 72 h after the transfection. **K** Measurement of OCR transfected with si-NC and si-*NDUFS6* in RPMI-8226 and MM.1 S cells after 72 h. Data represent the mean ± SD. Experiments were performed in triplicate. * *p* < 0.05, ** *p* < 0.01, *** *p* < 0.001. n.s. not significant.

To further explore how PRMT1 regulates OXPHOS-related genes, we conducted m^6^A sequencing (m^6^A -seq) in MM.1 S cells transfected with si-NC or si-*PRMT1*. The m^6^A -seq data revealed that m^6^A peaks were distributed in 3’-untranslated (3’-UTR) and the coding region (CDS) (Fig. 4A), consistent with previous findings [20, 21]. As shown in Fig. 4C, the consensus motif GGAC was abundant within m^6^A sites in both NC and *PRMT1* KD cells. Similarly, GSEA analysis revealed significant enrichment of the OXPHOS pathway in line with RNA-seq data (Fig. 4B). Then, we combined the RNA-seq and m^6^A -seq to identify the downstream target genes. Interestingly, we found that the mRNA level of *NDUFS6* decreased and its m^6^A peak increased in *PRMT1* KD cells (Fig. 4D). In line with m^6^A -seq data, MeRIP-qPCR indicated that knockdown of *PRMT1* significantly increased the m^6^A modification levels of *NDUFS6* mRNA (Fig. 4E). Consistently, qRT-PCR and western blotting results confirmed the decreased expression of NDUFS6 following *PRMT1* knockdown in MM.1 S and NCI-H929 cells and increased expression of NDUFS6 following PRMT1 overexpression in MM.1 S and RPMI-8226 cells (Fig. 4F, Supplementary Fig. 5A). Then, immunohistochemical analysis showed that NDUFS6 positive cells were decreased in tumors following *PRMT1* knockout (Supplementary Fig. 3B). Moreover, we found *NDUFS6* expression was significantly elevated in MM patients and exhibited an expression pattern similar to *PRMT1*(Fig. 4G, Supplementary Fig. 5B-D). In addition, we confirmed NDMM patients had higher NDUFS6 mRNA (*p* < 0.0001, Fig. 4H) and protein (Fig. 4I) levels compared with normal donors. Next, loss-of-function studies were performed to explore NDUFS6’s role in MM. The silenced efficiency was examined by qRT-PCR and western blotting in MM.1 S and RPMI-8226 cells (Supplementary Fig. 5E). As expected, silencing *NDUFS6* could significantly increase cellular ROS levels (Fig. 4J). In addition, OCR results showed decreased OCR levels in both RPMI-8226 and MM.1 S cells after transfection with si-*NDUFS6* (Fig. 4K). These results collectively confirmed NDUFS6 as a downstream effector of PRMT1, further emphasizing its functional significance in MM.

### PRMT1 induced NDUFS6 m^6^A modification through methylation of WTAP

**Fig. 5:**
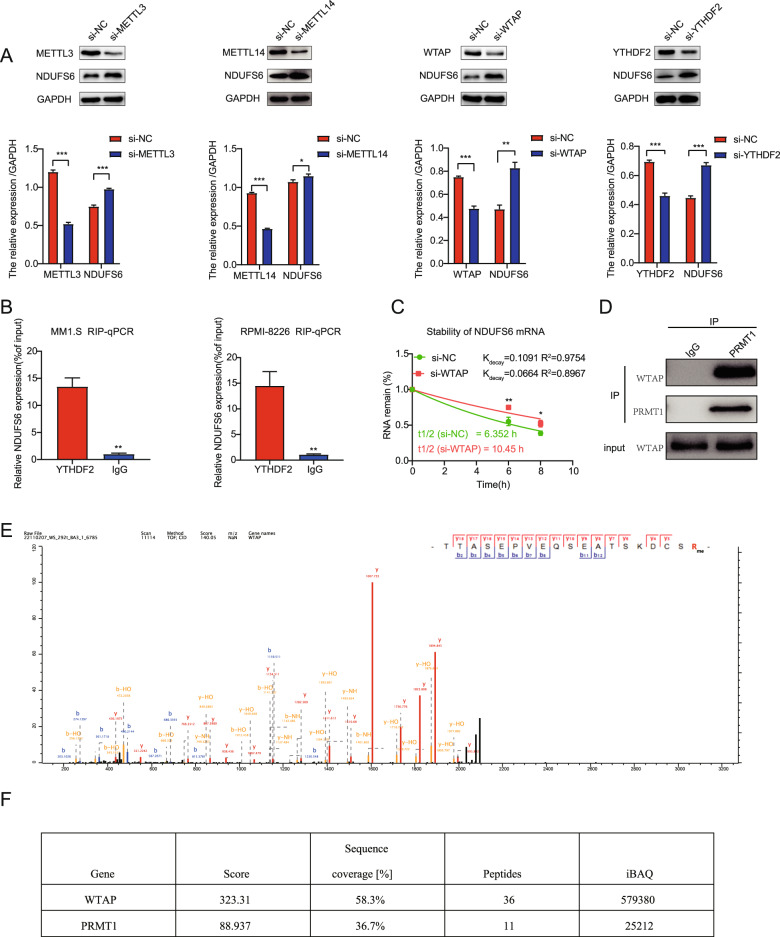
PRMT1 induced NDUFS6 m^6^A modification through methylation of WTAP. **A** Western blotting analysis of NDUFS6 protein level in NC and *METTL3* KD, *METTL14* KD, *WTAP* KD, and *YTHDF2* KD cells after 72 h following the transfection. **B** YTHDF2 immunoprecipitation assays of NDUFS6 in YTHDF2-bound mRNAs in MM.1 S and RPMI-8226 cells. **C** mRNA half-life (t1/2) of *NDUFS6* after actinomycin D treatment for 0, 6, and 8 h in NC and *WTAP* KD MM.1 S cells. **D** Co-IP showing the interaction of WTAP with IgG or PRMT1 in 293 T cells. **E** Mass spectrometry showing that WTAP R272 was methylated. **F** The score and sequence coverage (%) of WTAP and PRMT1 were identified by LC-MS/MS. Data represent the mean ± SD. Experiments were performed in triplicate. * *p* < 0.05, ** *p* < 0.01, *** *p* < 0.001.

Our previous results showed that *NDUFS6* m^6^A level was increased and mRNA level was decreased following *PRMT1* knockdown in MM cells. Next, we further confirmed that NDUFS6 expression was elevated by knocking down m^6^A writers, including METTL3, METTL14, and WTAP (Fig. 5A). YTH domain-containing family protein 2 (YTHDF2) is a major m^6^A reader to recognize its m^6^A modification [22]. Next, we found that NDUFS6 expression was increased after YTHDF2 knockdown in RPMI-8226 cells (Fig. 5A). Meanwhile, RIP-qPCR indicated that the expression of *NDUFS6* was increased by more than 10-fold after treatment with the YTHDF2 antibody in MM.1 S and RPMI-8226 cells compared with IgG antibody (Fig. 5B), which confirmed that m^6^A modulators regulated NDUFS6. In addition, we found that the half-life of *NDUFS6* mRNA was significantly prolonged in *WTAP* knockdown MM cells (6.352 h versus 10.45 h, *p* = 0.005, *p* = 0.014; Fig. 5C), which suggested that m^6^A modification accelerated the degradation of *NDUFS6* mRNA. Recent research revealed that PRMT1 could interact with and methylate METTL14 at R255 [23]. Accordingly, we performed Co-IP experiments which showed WTAP was the substrate of PRMT1 (Fig. 5D). To determine the methylation sites, tag-WTAP was subjected to LC-MS/MS. We found the R272 site of WTAP was monomethylated (Fig. 5E). Besides, we found PRMT1 was abundant in the pulled-down protein samples (Fig. 5F). It has been reported that protein arginine methylation regulates protein nuclear-cytoplasmic shuttling [24, 25]. However, we found the WTAP protein levels remained unchanged in total protein, nuclear, and cytoplasmic protein levels after *PRMT1* silencing (Supplementary Fig. 6). The results indicated that PRMT1 regulated the activity of WTAP but not the expression level through arginine methylation. Taken together, our findings suggested that PRMT1 could catalyze arginine methylation of WTAP, and NDUFS6 was identified as a bona fide m^6^A target of WTAP.

### PRMT1 induced activation of oxidative phosphorylation through the WTAP-NDUFS6 axis

**Fig. 6:**
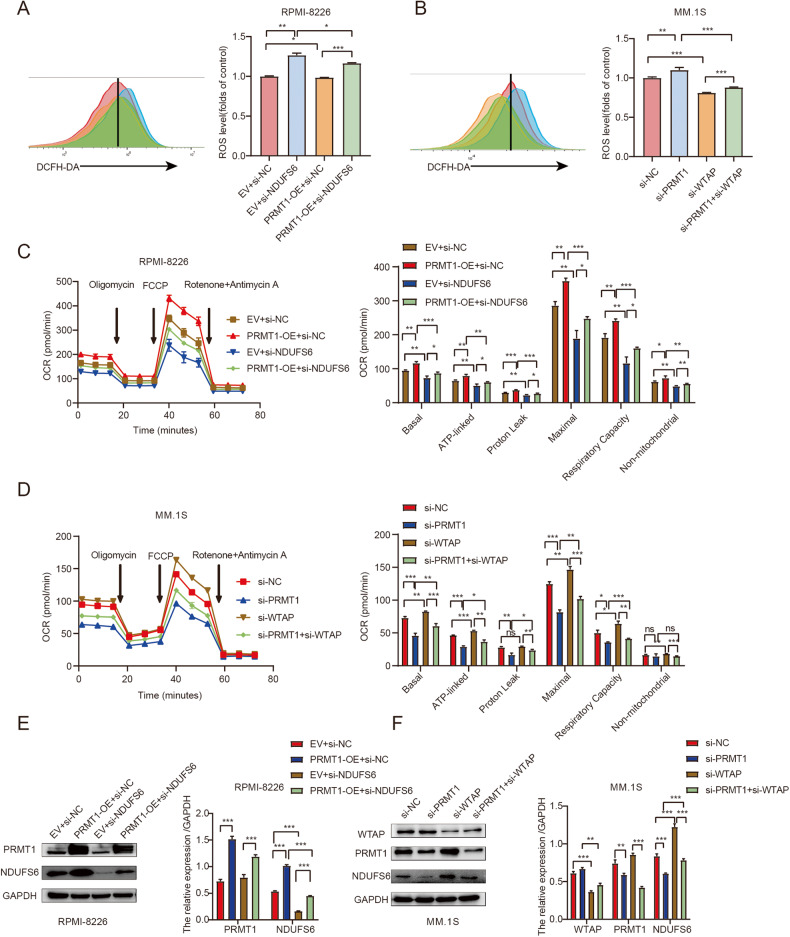
PRMT1 induced activation of oxidative phosphorylation through WTAP-NDUFS6 axis. **A** ROS level was assessed by flow cytometry in RPMI-8226 cells transfected with EV+si-NC, EV+si-*NDUFS6*, PRMT1-OE+si-NC, and PRMT1-OE+si-*NDUFS6* after 72 h. **B** ROS level was assessed by flow cytometry in MM.1 S cells transfected with si-NC, si-*PRMT1*, si-*WTAP*, and si-*PRMT1*+si-*WTAP* after 72 h. **C** Measurement of OCR in RPMI-8226 cells transfected with EV+si-NC, PRMT1-OE+si-NC, EV+si-*NDUFS6*, and PRMT1-OE+si-*NDUFS6* after 72 h. **D** Measurement of OCR transfected with si-NC, si-*PRMT1*, si-*WTAP*, and si-*PRMT1*+ si-*WTAP* in MM.1 S after 72 h. **E** Western blotting analysis of NDUFS6 protein level in RPMI-8226 cells transfected with EV+si-NC, PRMT1-OE+si-NC, EV+si-*NDUFS6*, and PRMT1-OE+si-*NDUFS6* after 72 h. **F** Western blotting analysis of NDUFS6 protein level in MM.1 S cells transfected with si-NC, si-*PRMT1*, si-*WTAP*, and si-*PRMT1*+ si-*WTAP* after 72 h. Data are expressed as the mean ± SD. Experiments were performed in triplicate. * *p* < 0.05, ** *p* < 0.01, *** *p* < 0.001. n.s. not significant.

To confirm whether PRMT1 regulates oxidative phosphorylation through the WTAP-NDUFS6 axis, we conducted experiments to examine the effects of silencing *WTAP* or *NDUFS6* after PRMT1 downregulation or upregulation on ROS levels. We found that *NDUFS6* knockdown could reverse the decrease in ROS levels induced by PRMT1 overexpression (Fig. 6A). Consistently, silencing *WTAP* induced a decrease in ROS levels and counter the increase in ROS levels induced by *PRMT1* silencing (Fig. 6B). To further validate the role of PRMT1-WTAP-NDUFS6 in MM, OCR levels were examined. Knockdown of *NDUFS6* could counter the increase in OCR induced by PRMT1 overexpression (Fig. 6C). Meanwhile, *WTAP* knockdown increased the OCR levels and could reverse the decrease in OCR induced by *PRMT1* knockdown (Fig. 6D). Finally, we observed that silencing *NDUFS6* could reverse the increase in protein expression of NDUFS6 induced by PRMT1 overexpression and silencing *WTAP* could counter the decrease in protein expression of NDUFS6 induced by *PRMT1* knockdown (Figs. 6E and 6F). Taken together, our findings further supporte that the dysregulation of oxidative phosphorylation caused by PRMT1 is at least partially through WTAP-NDUFS6 axis.

### Synergistic cytotoxic effect of combined PRMT1 inhibitor and BTZ in MM

**Fig. 7:**
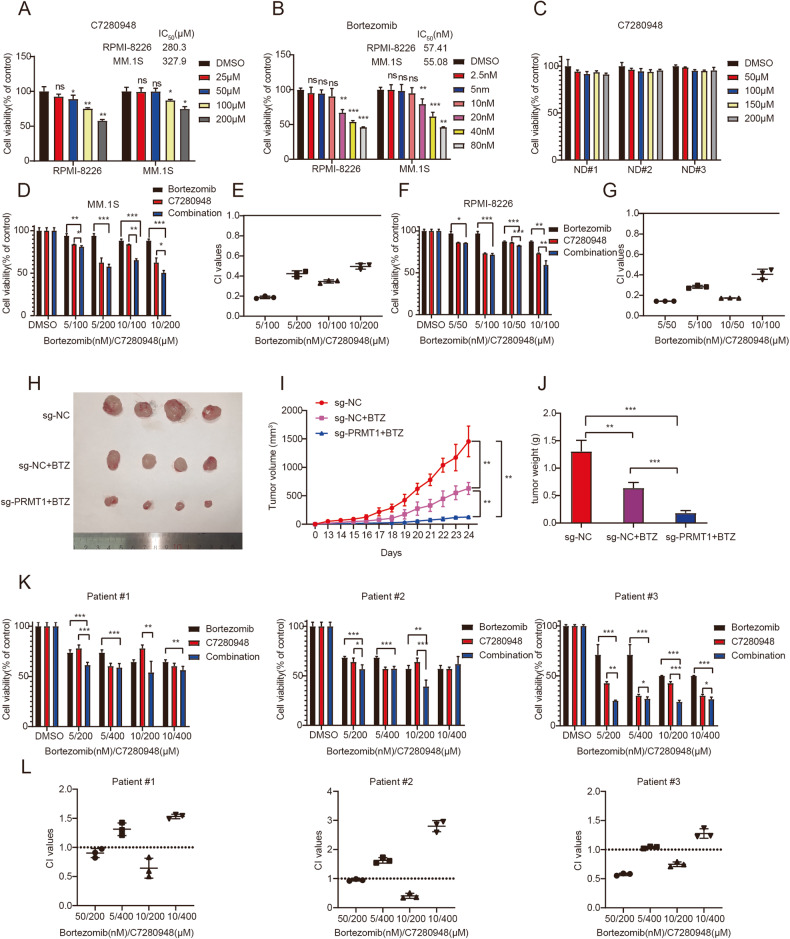
Synergistic cytotoxic effect of combined PRMT1 inhibitor and BTZ in MM. **A** Cell viability of MM cell lines (RPMI-8226 and MM.1S) treated with C7280948 for 48 h. **B** Cell viability of MM cell lines (RPMI-8226 and MM1.S) treated with BTZ for 48 h. **C** Cell viability of freshly peripheral blood mononuclear cells (PBMCs) from 3 healthy donors were cultured with 50 μM-200μM of C7280948 for 48 h. **D** Cell viability of MM.1 S cells treated with C7280948 and BTZ, alone or in combination for 48 h. **E** CI values of MM.1 S cells treated for 48 h with C7280948 and BTZ, alone or in combination, were calculated using CompuSyn. **F** Cell viability of RPMI-8226 cells treated with C7280948 and BTZ, alone or in combination for 48 h. **G** CI values of RPMI-8226 cells treated for 48 h with C7280948 and BTZ, alone or in combination, were calculated using CompuSyn. **H** Photographs of tumors. **I** Quantification of the tumor volumes in nude mice after implantation of MM cells (mean tumor volume±S.D., 4 mice per group). **J** Quantification of tumor weights from MM-xenograft mice on day 24. **K** Viability of CD138^+^ cells isolated from three NDMM patients treated with C7280948 and BTZ, alone or in combination for 48 h, respectively. **L** CI values of CD138^+^ cells isolated from three NDMM patients treated for 48 h with C7280948 and BTZ, alone or in combination, were calculated using CompuSyn. DMSO in this figure represents vehicle control (0.1% DMSO). Data represent the mean ± SD. Experiments were performed in triplicate. * *p* < 0.05, ** *p* < 0.01, *** *p* < 0.001. n.s. not significant.

To further evaluate the potential clinical value of PRMT1 in the treatment of MM, we selected the PRMT1-selective small-molecule inhibitor C7280948, which has been reported to suppress cell proliferation, migration, and invasion in colorectal cancer [26]. Our results showed that either C7280948 or BTZ significantly reduced the viability of MM cell lines in a dose-dependent manner (Fig. 7A-B) but without an obvious effect on blood mononuclear cells (PBMCs) derived from 3 normal healthy donors (Fig. 7C). Next, we combined C7280948 and BTZ to treat MM cells. We found that the combination of C7280948 and BTZ synergistically abrogated the growth of MM in both MM.1S and RPMI-8226 cells (Fig. 7D, F), with all combination index values < 1 (Fig. 7E, G), indicating a synergistic effect between C7280948 and BTZ. Meanwhile, the combination of BTZ and sg-PRMT1 resulted in significant tumor inhibition in a xenograft MM mouse model (Fig. 7H-J). Moreover, we isolated CD138^+^ cells from 3 NDMM patients and treated cells with C7280948 and BTZ. As illustrated in Fig. 7K-L, combination index values < 1 were observed when 200 μM C7280948 was used. Simultaneous treatment of CD138^+^ cells with C7280948 and BTZ resulted in significantly higher apoptosis levels (Supplementary Fig. 7). Our results showed that C7280948 could increase the sensitivity of MM cells to BTZ. Taken together, these results indicated that the combination of BTZ treatment and PRMT1 inhibition yielded synergistic cytotoxic effects in both MM cell lines and primary MM patient cells.

## Discussion

MM is the second most common hematological malignancy and poses significant challenges due to its incurable nature, attributed to drug resistance and replapse [7]. Accordingly, understanding the pathogenesis and exploring novel treatment targets are paramount for MM. Epigenetic modifications, such as arginine methylation, are reportedly involved in MM progression. PRMT5, a type II arginine methyltransferase, was upregulated in MM and associated with worse PFS and OS. PRMT5 inhibition has been shown to induce cell autophagy through the TRIM21/IKKB pathway [27]. Xia et al. [28] reported that PRMT5 could regulate cell pyroptosis via silencing CASP1 in MM.

PRMT1, a type I arginine methyltransferase, has been reported to play an important role in the progression of various diseases, especially hematological diseases. Existing studies on PRMT1 have mostly focused on leukemia. Recent studies have shown that RNA binding protein RBM15 could be methylated by PRMT1, which resulted in RBM15 degradation via ubiquitylation and retardation of megakaryocyte differentiation in acute megakaryocytic leukemia (AMKL). Accordingly, PRMT1 may be a potential target for treating AMKL [29]. A study by Xin He et al. [30] revealed that increased PRMT1 catalyzed FLT3-ITD protein methylation, leading to acute myeloid leukemia (AML) cell growth. Combining the tyrosine kinase inhibitor (TKI) AC220 with a PRMT1 inhibitor (MS023) enhanced the cytotoxic effect of FLT3-ITD + AML cells. In addition, Lei et al. [31] demonstrated that PRMT1 is necessary for maintaining normal adult hematopoiesis. Besides, high PRMT1 expression has been associated with adverse outcomes in myelodysplastic syndrome (MDS). Interestingly, it has been shown that inhibition of PRMT1 could stimulate megakaryopoiesis [32]. A study found that PRMT1 inhibitors could pharmacologically target γ-globin suppression in β-hemoglobinopathies [33]. Besides, PRMT1 has been associated with glucocorticoid resistance in acute lymphoblastic leukemia [34]. However, few studies have focused on the role of PRMT1 in MM. To our knowledge, this is the first study to reveal that PRMT1 is upregulated in MM patients. In addition, the expression of *PRMT1* was positively correlated with the ISS and R-ISS stages. Multivariate Cox regression analysis revealed that *PRMT1* expression was an independent adverse prognostic factor for OS in MM. Moreover, we constructed a nomogram model based on *PRMT1* expression, which could predict MM patient survival accurately. These findings suggested that *PRMT1* acts as an oncogene in MM. Next, we further demonstrated that silencing *PRMT1* decreased MM cell proliferation and increased cell apoptosis in vitro. Loss of *PRMT1* restrained tumor growth in vivo. Consistently, Li et al. [16] demonstrated that PRMT1 could promote breast cancer cell proliferation. Lee et al. [35] found that ROS levels were slightly increased in *PRMT1* KD SK-N-SH cells. Furthermore, we identified the OXPHOS pathway as a target of PRMT1 by analyzing RNA-seq data and MeRIP-seq data based on the pathway enrichment. Interestingly, we found that most of the OXPHOS genes were significantly decreased in *PRMT1* KD cells. TEM imaging and OCR results verified our hypothesis that silencing *PRMT1* caused OXPHOS dysfunction and OXPHOS predominantly generated ROS in mitochondria. Increased ROS levels in *PRMT1* KD cells confirmed the dysregulation of OXPHOS.

According to Warburg [36], cancer cells may use glucose via glycolysis over OXPHOS, despite oxygen availability. However, there is growing evidence that genes involved in OXPHOS are upregulated in cancers [37, 38]. A recent study reported that MM relies on enhanced OXPHOS, although described as glycolysis-dependent in previous studies [39]. NDUFS6 is a crucial subunit in mitochondrial complex I. Deficiency of NDUFS6 has been reported to reduce complex I mitochondrial function leading to OXPHOS disorders [40]. In our study, NDUFS6 expression was higher in MM patients than in normal donors. Besides, MM patients with higher expression of *NDUFS6* were notably related to adverse OS. High expression of *NDUFS6* was positively correlated with MM stages. In addition, we explored the function of NDUFS6 in MM. As expected, silencing *NDUFS6* significantly increased cellular ROS levels and decreased OCR levels in MM cells. *NDUFS6* knockdown could reverse ROS levels and the OCR levels caused by PRMT1 overexpression. The above findings indicated that NDUFS6 is a downstream effector of PRMT1 in MM.

MeRIP-seq data indicated that *NDUFS6* m^6^A peak increased in *PRMT1* KD cells. Accordingly, we further knocked down m^6^A writers, including METTL3, METTL14, and WTAP. The results of our study showed that the knockdown of m^6^A writers led to an increase in NDUFS6 protein expression. Next, the Co-IP results revealed that WTAP could be the bridge connecting PRMT1 and NDUFS6. We found that PRMT1 regulated the ADMA level of WTAP (data not shown), which led to the m^6^A peak and NDUFS6 expression changes. Interestingly, *WTAP* knockdown could reverse OCR levels, ROS levels, and the protein level of NDUFS6 upon *PRMT1* knockdown. Taken together, these data confirmed the function of the PRMT1-WTAP-NDUFS6 pathway in MM progression. Consistently, ALKBH5 [41], FTO [42], METTL3 [43], and YTHDF2 [44] have been reported to play a crucial role in MM, highlighting the important function of m^6^A in MM.

A promising proteasome inhibitor, BTZ, is currently used to treat multiple myeloma. However, relapses are frequent, and resistance to proteasome inhibitors treatment is a major clinical problem in most MM patients [45]. It is crucial to identify new, more effective drugs for MM. C7280948 is a specific inhibitor for PRMT1, which could suppress colorectal cancer progression [17]. Our study found that C7280948 reduced the viability of MM cell lines and CD138^+^ cells from MM patients in a concentration-dependent manner. On the other hand, C7280948 could increase the sensitivity of MM cells to BTZ. However, the IC_50_ of C7280948 in vitro is too high to apply to mice. So,we conducted an in vivo experiment by subcutaneously injecting *PRMT1* knockout MM.1 S cells into nude mice. Subsequently, we administered BTZ intraperitoneally to assess the synergistic effect. Interestingly, the combination of knocking out *PRMT1* and BTZ resulted in a stronger inhibition than BTZ alone in vivo. The specific mechanisms underlying this synergistic effect required further investigation. Overall, our findings provided a theoretical basis for treating MM patients with PRMT1 inhibitors and BTZ.

In conclusion, our data demonstrated that PRMT1 enhanced MM cell proliferation and OCR levels and decreased cell apoptosis and ROS levels by binding with WTAP to regulate NDUFS6 expression via an m^6^A-dependent manner. Importantly, the PRMT1 inhibitor could increase the sensitivity of MM cells to BTZ. These findings provide a novel strategy and potential target for MM therapy in the future.

## Supplementary information

Supplementary Material

Original Data File

CDD Checklist

## Data Availability

All data included in this study are available from the corresponding authors upon reasonable request.

## References

[1] Kumar SK, Rajkumar V, Kyle RA, Duin MV, Anderson KC (2017). Multiple myeloma. Nat Rev Dis Primers.

[2] Sisay M, Barac A, Bensenor I, Curado MP, Tran B. Global Burden of Multiple Myeloma: A Systematic Analysis for the Global Burden of Disease Study 2016. Jama Oncol 2018. 10.1001/jamaoncol.2018.2128 PMC6143021 29800065

[3] Natural history of relapsed myeloma, refractory to immunomodulatory drugs and proteasome inhibitors: a multicenter IMWG study. Leukemia 2017. 10.1038/leu.2017.138 28620163

[4] Rajkumar SV, Kumar S Multiple myeloma current treatment algorithms. Blood Cancer J 2020;10. 10.1038/s41408-020-00359-2 PMC7523011 32989217

[5] Zhao WH, Wang BY, Chen LJ, Fu WJ, Xu J, Liu J, et al. Four-year follow-up of LCAR-B38M in relapsed or refractory multiple myeloma: a phase 1, single-arm, open-label, multicenter study in China (LEGEND-2). J Hematol Oncol 2022;15. 10.1186/s13045-022-01301-8 PMC9261106 35794616

[6] Pinto V, Bergantim R, Caires HR, Seca H, Guimaraes JE, Vasconcelos MH. Multiple Myeloma: Available Therapies and Causes of Drug Resistance. Cancers. 2020;12 10.3390/cancers12020407 PMC7072128 32050631

[7] Robak P, Drozdz I, Szemraj J, Robak T (2018). Drug resistance in multiple myeloma. Cancer Treat Rev.

[8] Corre J, Munshi N, Avet-Loiseau H (2015). Genetics of multiple myeloma: another heterogeneity level?. Blood.

[9] Bianchi G, Munshi NC (2015). Pathogenesis beyond the cancer clone(s) in multiple myeloma. Blood.

[10] De Smedt E, Maes K, Verhulst S, Lui H, Kassambara A, Maes A (2018). Loss of RASSF4 Expression in Multiple Myeloma Promotes RAS-Driven Malignant Progression. Cancer Res.

[11] Hu H, Qian K, Ho MC, Zheng YG (2016). Small Molecule Inhibitors of Protein Arginine Methyltransferases. Expert Opin Investig Drugs.

[12] Blanc RS, Richard S (2017). Arginine Methylation: The Coming of Age. Mol Cell.

[13] Tang J, Frankel A, Cook RJ, Kim S, Paik WK, Williams KR (2000). PRMT1 is the predominant type I protein arginine methyltransferase in mammalian cells. J Biol Chem.

[14] Yoshimatsu M, Toyokawa G, Hayami S, Unoki M, Tsunoda T, Field HI (2011). Dysregulation of PRMT1 and PRMT6, Type I arginine methyltransferases, is involved in various types of human cancers. Int J Cancer.

[15] Avasarala S, Van Scoyk M, Rathinam MKK, Zerayesus S, Zhao XM, Zhang W (2015). PRMT1 Is a Novel Regulator of Epithelial-Mesenchymal-Transition in Non-small Cell Lung Cancer. Journal of Biological Chemistry.

[16] Li ZW, Wang DD, Lu J, Huang BQ, Wang YB, Dong MC (2020). Methylation of EZH2 by PRMT1 regulates its stability and promotes breast cancer metastasis. Cell Death Differ.

[17] Yin XK, Wang YL, Wang F, Feng WX, Bai SM, Zhao WW (2021). PRMT1 enhances oncogenic arginine methylation of NONO in colorectal cancer. Oncogene.

[18] Zhang B, Wu Q, Li B, Wang DF, Wang L, Zhou YL. m(6)A regulator-mediated methylation modification patterns and tumor microenvironment infiltration characterization in gastric cancer. Mol Cancer 2020;19. 10.1186/s12943-020-01170-0 PMC7066851 32164750

[19] Alirol E, Martinou JC (2006). Mitochondria and cancer: is there a morphological connection?. Oncogene.

[20] Meyer KD, Saletore Y, Zumbo P, Elemento O, Mason CE, Jaffrey SR. Comprehensive analysis of mRNA methylation reveals enrichment in 3’ UTRs and near stop codons. Cell. 2012. 10.1016/j.cell.2012.05.003 PMC3383396 22608085

[21] Dominissini D, Moshitch-Moshkovitz S, Schwartz S, Salmon-Divon M, Ungar L, Osenberg S (2012). Topology of the human and mouse m6A RNA methylomes revealed by m6A-seq. Nature.

[22] Wang X, Lu Z, Gomez A, Hon GC, Yue Y, Han D (2014). N6-methyladenosine-dependent regulation of messenger RNA stability. Nature.

[23] Liu X, Wang H, Zhao X, Luo Q, Xiao S Arginine methylation of METTL14 promotes RNA N6-methyladenosine modification and endoderm differentiation of mouse embryonic stem cells. Nat Commun. 2021;12. 10.1038/s41467-021-24035-6 PMC8213825 34145242

[24] Green DM, Marfatia KA, Crafton EB, Zhang X, Cheng XD, Corbett AH (2002). Nab2p is required for poly(A) RNA export in Saccharomyces cerevisiae and is regulated by arginine methylation via Hmt1p. J Biol Chem.

[25] Aoki K, Ishii Y, Matsumoto K, Tsujimoto M (2002). Methylation of Xenopus CIRP2 regulates its arginine- and glycine-rich region-mediated nucleocytoplasmic distribution. Nucleic Acids Res.

[26] Yin XK, Wang YL, Wang F, Feng WX, Wan XB. PRMT1 enhances oncogenic arginine methylation of NONO in colorectal cancer. Oncogene 2021:1–15. 10.1038/s41388-020-01617-0 PMC7892343 33420374

[27] Gullà A, Hideshima T, Bianchi G, Fulciniti M, Samur MK, Qi J, et al. Protein Arginine Methyltransferase 5 (PRMT5) has prognostic relevance and is a druggable target in Multiple Myeloma. Leukemia 2017. 10.1038/leu.2017.334 PMC5871539 29158558

[28] Xia T, Liu M, Zhao Q, Zhou RF, Chen B, Xu PP PRMT5 Regulates Cell Pyroptosis By Silencing CASP1 in Multiple Myeloma. Blood 2021;138. 10.1038/s41419-021-04125-5 PMC8445991 34531375

[29] Zhang L, Tran NT, Su HR, Wang R, Lu YH, Tang HP, et al. Cross-talk between PRMT1-mediated methylation and ubiquitylation on RBM15 controls RNA splicing. Elife 2015;4. 10.7554/eLife.07938 PMC4775220 26575292

[30] He X, Zhu YH, Lin YC, Li M, Du J, Dong HJ (2019). PRMT1-mediated FLT3 arginine methylation promotes maintenance of FLT3-ITD+ acute myeloid leukemia. Blood.

[31] Zhu L, He X, Dong HJ, Sun J, Wang HY, Zhu YH (2019). Protein arginine methyltransferase 1 is required for maintenance of normal adult hematopoiesis. Int J Biol Sci.

[32] Su HR, Jiang M, Senevirathne C, Aluri S, Zhang T, Guo H, et al. Methylation of dual-specificity phosphatase 4 controls cell differentiation. Cell Rep 2021;36. 10.1016/j.celrep.2021.109421 PMC9110119 34320342

[33] Wang Y, Li X, Ge J, Liu M, Pang X, Liu J (2021). The methyltransferase PRMT1 regulates gamma-globin translation. J Biol Chem.

[34] van Galen JC, Kuiper RP, van Emst L, Levers M, Tijchon E, Scheijen B (2010). BTG1 regulates glucocorticoid receptor autoinduction in acute lymphoblastic leukemia. Blood.

[35] Lee YJ, Chang WW, Chang CP, Liu TY, Chuang CY, Qian K, et al. Downregulation of PRMT1 promotes the senescence and migration of a non-MYCN amplified neuroblastoma SK-N-SH cells. Sci Rep-Uk. 2019;9. 10.1038/s41598-018-38394-6 PMC6370813 30741995

[36] Warburg O (1956). On the origin of cancer cells. Science.

[37] Ashton TM, Mckenna WG, Kunz-Schughart LA, Higgins GS Oxidative phosphorylation as an emerging target in cancer therapy. Clin Cancer Res 2018: clincanres.3070.2017. 10.1158/1078-0432.CCR-17-3070 29420223

[38] Kong G, You X, Wen Z, Chang Y, Qian S, Ranheim EA, et al. Downregulating Notch Counteracts KrasG12D-Induced ERK Activation and Oxidative Phosphorylation in Myeloproliferative Neoplasm. Leukemia. 10.1038/s41375-018-0248-0 PMC6405304 30206308

[39] Dalva-Aydemir S, Bajpai R, Martinez M, Adekola KUA, Kandela I, Wei CY (2015). Targeting the Metabolic Plasticity of Multiple Myeloma with FDA-Approved Ritonavir and Metformin. Clinical Cancer Research.

[40] Forbes JM, Ke BX, Nguyen TV, Henstridge DC, Penfold SA, Laskowski A (2013). Deficiency in Mitochondrial Complex I Activity Due to Ndufs6 Gene Trap Insertion Induces Renal Disease. Antioxid Redox Sign.

[41] Qu JW, Hou YF, Chen QX, Chen J, Li Y, Zhang EF (2022). RNA demethylase ALKBH5 promotes tumorigenesis in multiple myeloma via TRAF1-mediated activation of NF-kappa B and MAPK signaling pathways. Oncogene.

[42] Xu AS, Zhang JS, Zuo LP, Yan H, Chen L, Zhao F (2022). FTO promotes multiple myeloma progression by posttranscriptional activation of HSF1 in an m(6)A-YTHDF2-dependent manner. Mol Ther.

[43] Che FF, Ye XM, Wang Y, Wang XM, Ma SY, Tan YW, et al. METTL3 facilitates multiple myeloma tumorigenesis by enhancing YY1 stability and pri-microRNA-27 maturation in m(6)A-dependent manner. Cell Biol Toxicol 2022. 10.1007/s10565-021-09690-1 35038059

[44] Hua Z, Wei RF, Guo MJ, Lin ZG, Yu XC, Li XY (2022). YTHDF2 promotes multiple myeloma cell proliferation via STAT5A/MAP2K2/p-ERK axis. Oncogene.

[45] Qiang W, Zla B, Zhuo W, Ly A, Miao XA, Lx A, et al. RARγ activation sensitizes human myeloma cells to carfilzomib treatment through OAS-RNase L innate immune pathway. 2021. 10.1182/blood.2020009856 34411225

