# Supplementary material for: PRMT1 methylation of WTAP promotes multiple myeloma tumorigenesis by activating oxidative phosphorylation via m6A modification of NDUFS6
Source: Cell Death Dis. 2023 Aug 9;14(8):512. doi: 10.1038/s41419-023-06036-z (PMC10412649; doi:10.1038/s41419-023-06036-z)
Supplement: Supplementary file 1 — Supplementary Material [file 41419_2023_6036_MOESM1_ESM.docx]

Supplementary Material

**Supplementary Figure**


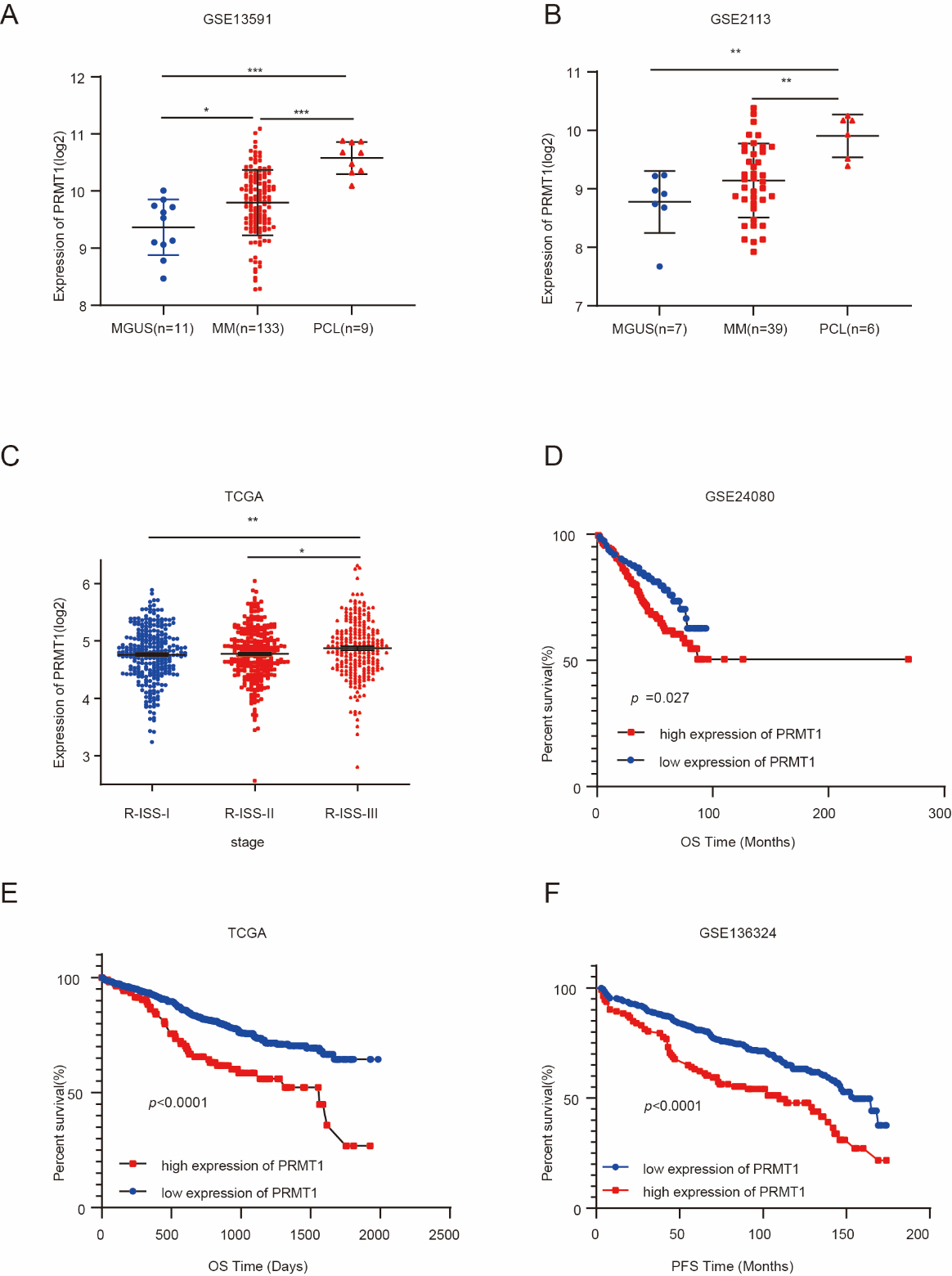


**Figure S1 Elevated PRMT1 expression was related to adverse outcomes in MM patients**

(A-C). *PRMT1* expression was analyzed in public datasets (GSE13591, GSE2113, and TCGA cohort). (D-F) Kaplan-Meier survival analysis of overall survival rate (D-E) and progression-free survival rate (F) in GSE24080, TCGA cohort, and GSE136324, respectively. Data represent the mean ± SD.* *p*<0.05, ** *p*<0.01, *** *p*<0.001.


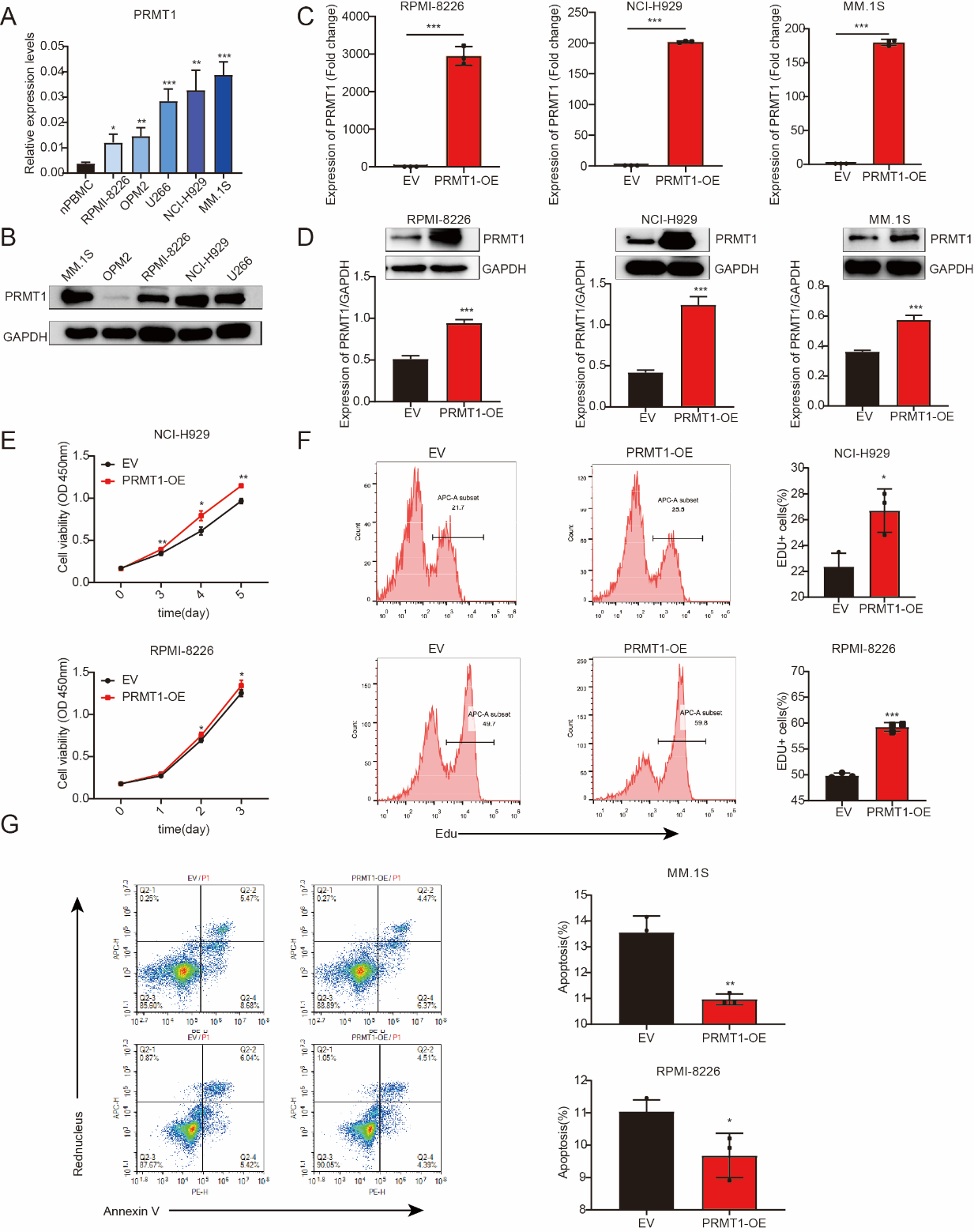


**Figure S2 Upregulation of PRMT1 enhanced MM proliferation**

(A) *PRMT1* mRNA expression was detected by qRT-PCR in normal peripheral blood mononuclear cells (nPBMC) and MM cell lines. (B) PRMT1 protein expression was detected by western blotting in MM cell lines. (C) The mRNA expression of *PRMT1* was detected by qRT-PCR in RPMI-8226, NCI-H929, and MM.1S cells transfected with EV or PRMT1-OE. (D) The protein expression of PRMT1 was detected by western blotting in RPMI-8226, NCI-H929, and MM.1S cells transfected with EV or PRMT1-OE. Proliferation of MM cells transfected with EV or PRMT1-OE, as determined by CCK8 (E) and EDU-staining (F). (G) Apoptotic analysis of MM cells by flow cytometry. Data represent the mean ± SD. Experiments were performed in triplicate. * *p*<0.05, ** *p*<0.01, *** *p*<0.001.


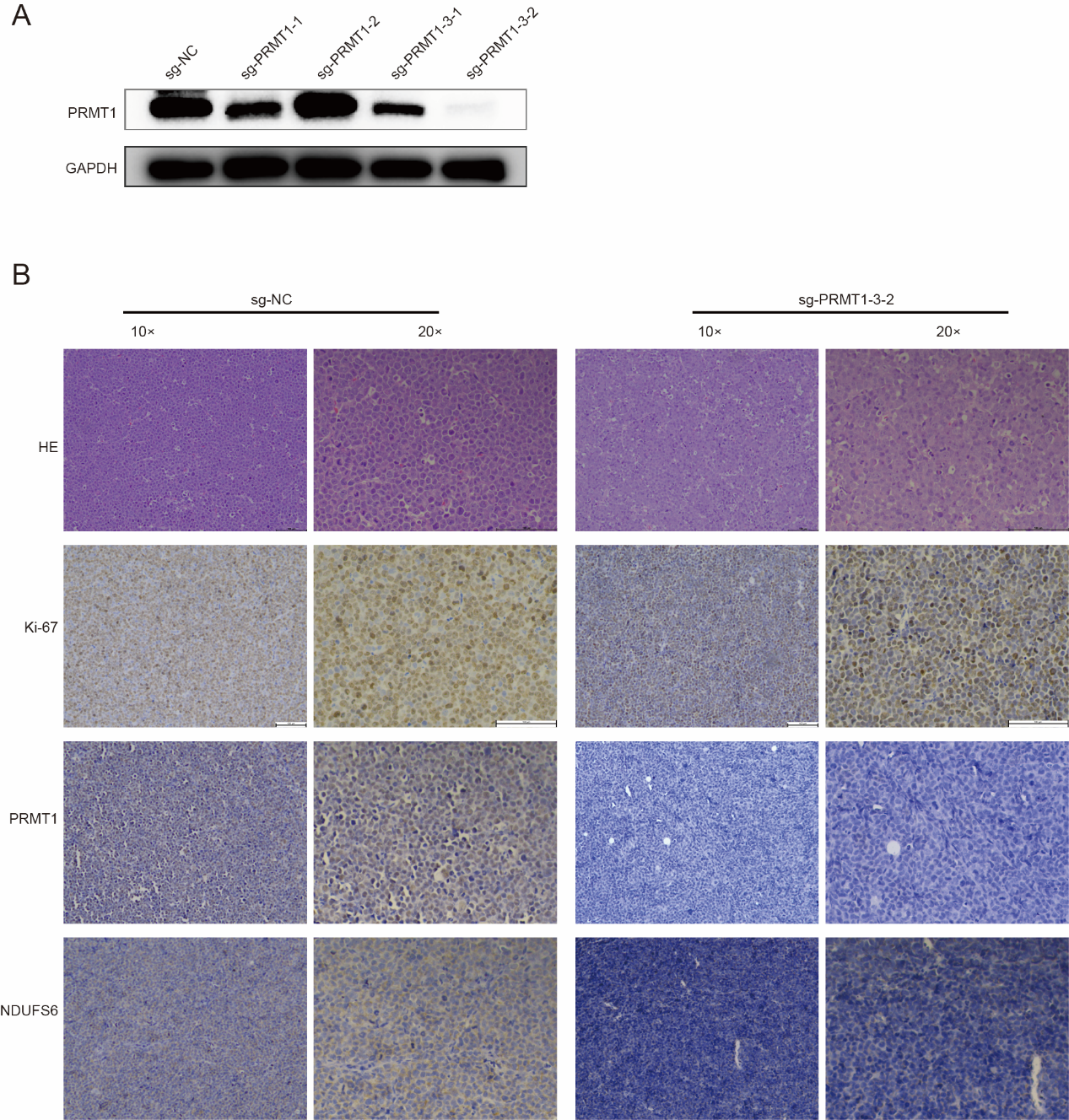


**Figure S3 Downregulation of PRMT1 inhibited MM progression *in vivo***

(A) PRMT1 protein expression was examined by western blotting in MM.1S cells transfected with sg-NC, sg-PRMT1-1, sg-PRMT1-2, sg-PRMT1-3-1, and sg-PRMT1-3-2. (B) H-E staining and immunohistochemical of Ki-67, PRMT1, and NDUFS6 in MM xenograft.


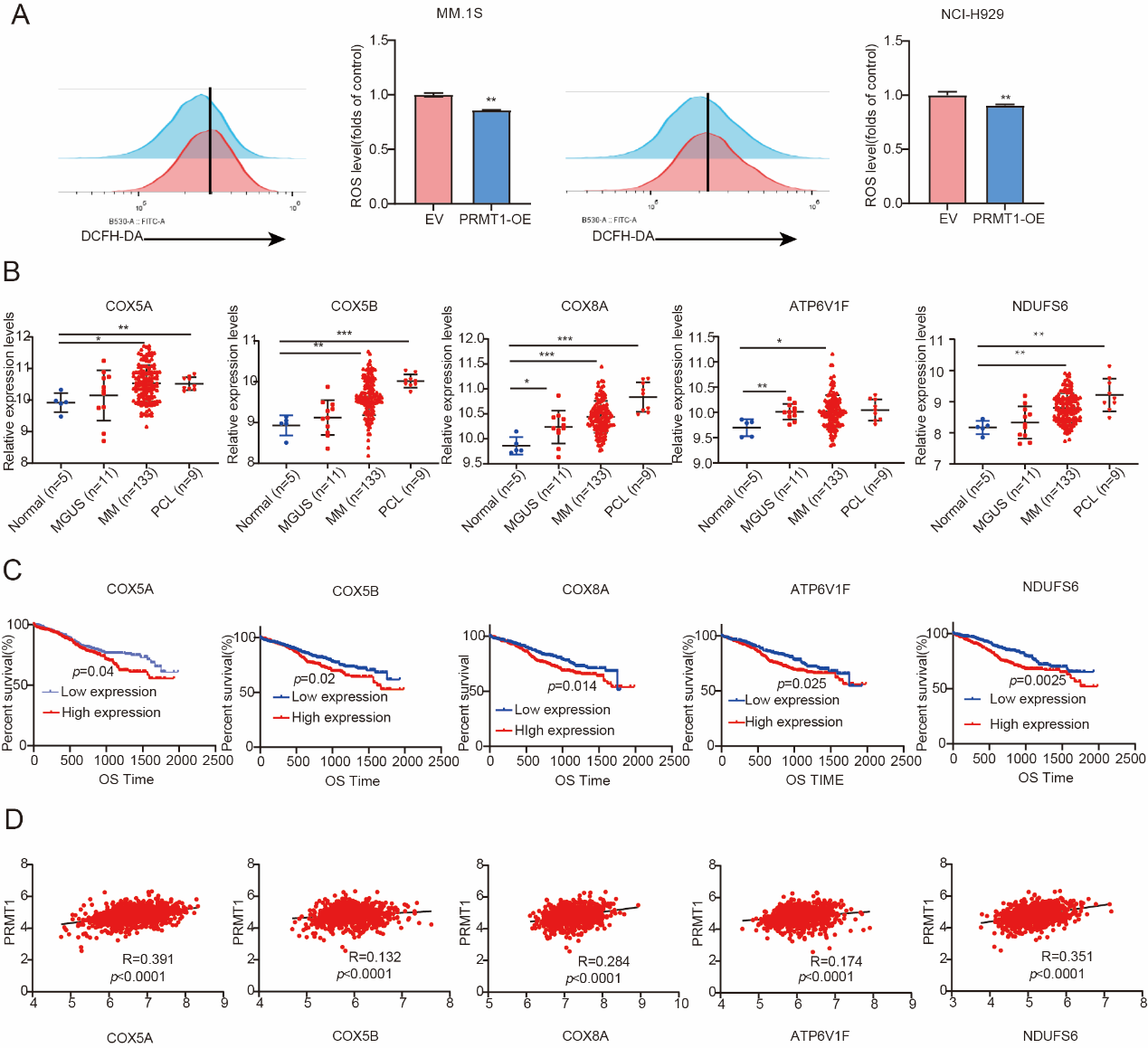


**Figure S4** **Downregulation of PRMT1 inhibited oxidative phosphorylation**

(A) ROS levels were assessed by flow cytometry in MM.1S and NCI-H929 cells transfected with EV and PRMT1-OE. (B) The expression of OXPHOS genes in MM progression processes: normal donor, MGUS, MM, and PCL in GSE13591. (C) Kaplan-Meier survival analysis of OS in TCGA cohort. (D) Pearson’s correlation between *PRMT1* and OXPHOS gene mRNA expression in TCGA cohort. Data represent the mean ± SD. Experiments were performed in triplicate. * *p*<0.05, ** *p*<0.01, *** *p*<0.001.


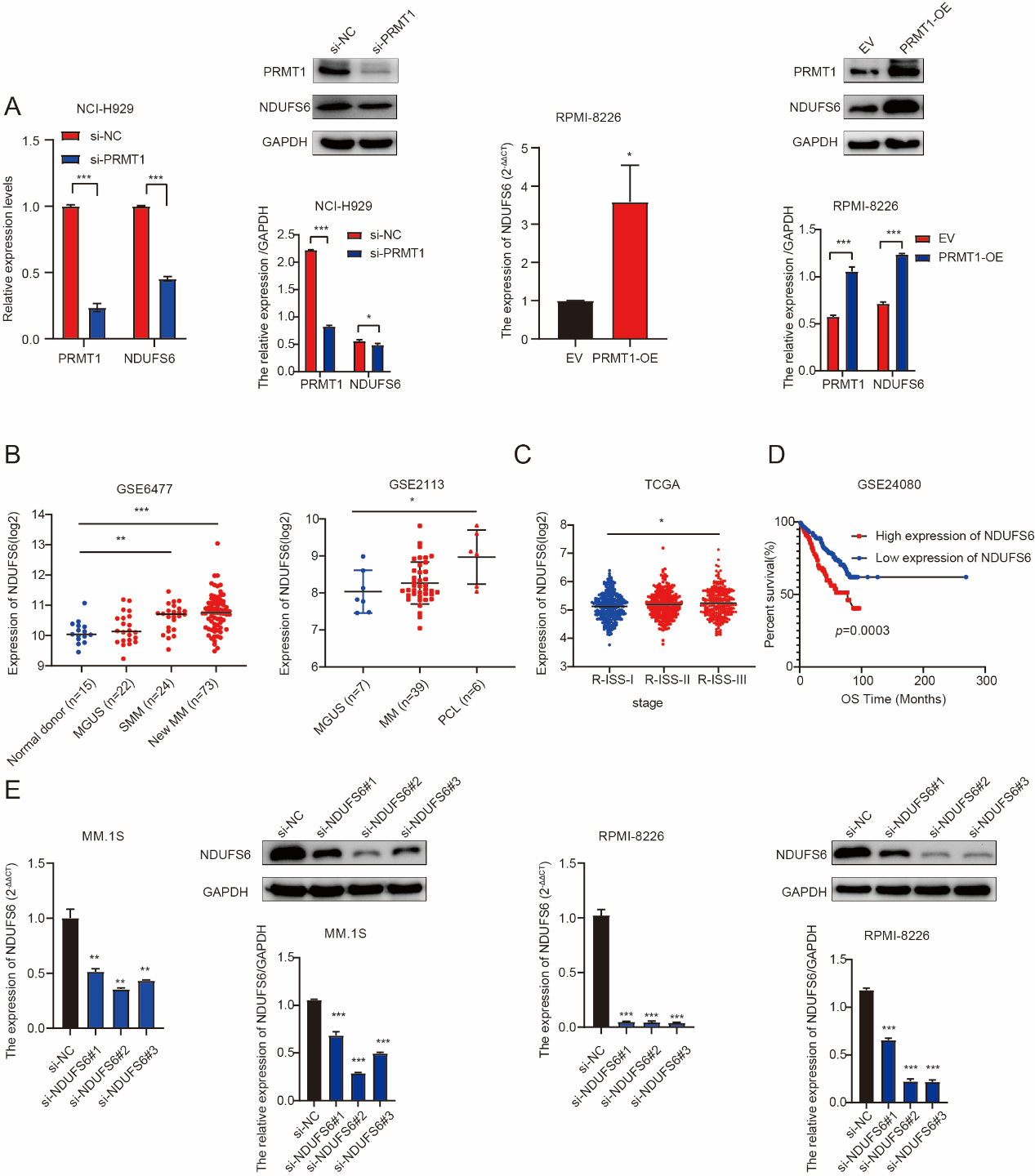


**Figure S5 NDUFS6 was the potential downstream effector of PRMT1 in MM**

(A) The mRNA and protein levels of NDUFS6 were verified by qRT-PCR and western blotting in NCI-H929 with or without *PRMT1* KD, and in RPMI-8226 cells with or without PRMT1-OE. (B) *NDUFS6* expression in MM progression processes: normal donor, MGUS, SMM and new MM in GSE6477; MGUS, MM, PCL in GSE2113. (C) *NDUFS6* expression in R-ISS stages in TCGA cohort. (D) Kaplan-Meier survival analysis of OS in GSE24080. (E) The mRNA and protein expression of NDUFS6 was verified by qRT-PCR and western blotting in MM.1S and RPMI-8226 cells with or without *NDUFS6* KD. Data represent the mean ± SD. Experiments were performed in triplicate. * *p*<0.05, ** *p*<0.01, *** *p*<0.001.


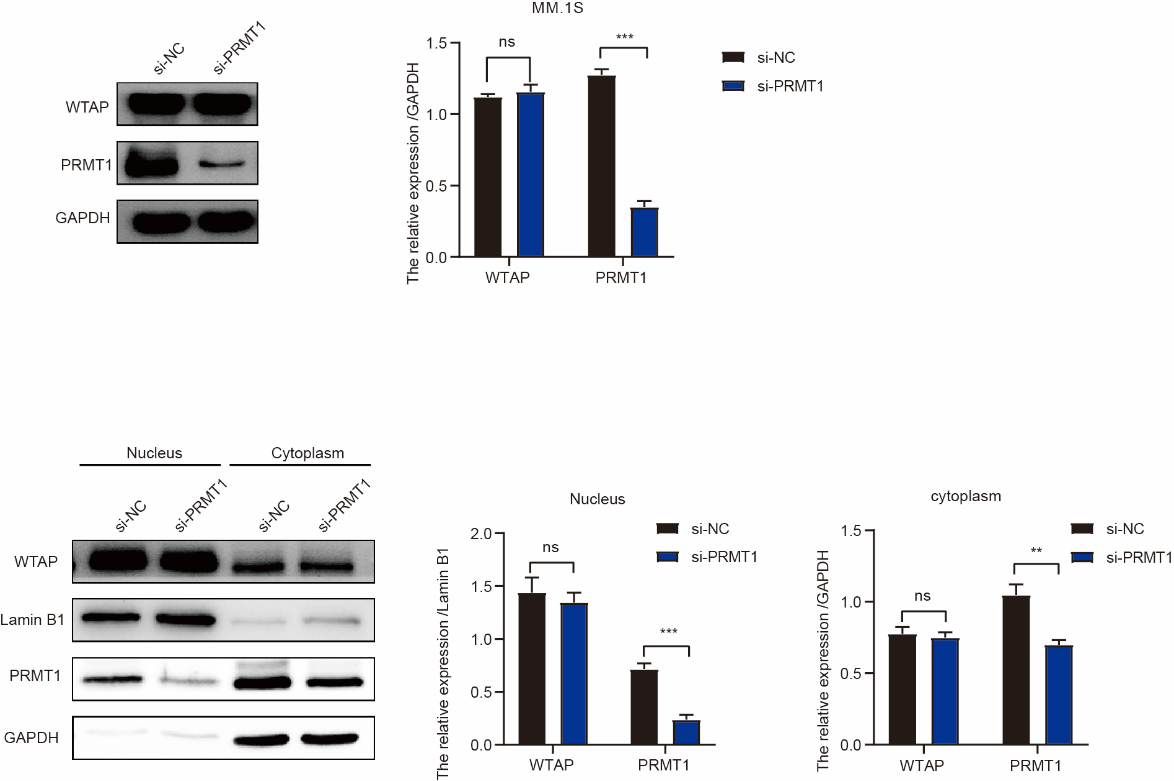


**Figure S6 PRMT1 induced NDUFS6 m^6^A modification through methylation of WTAP**

Western blotting showing the subcellular location of WTAP, and PRMT1 in 293T NC and PRMT1 KD cells. Data represent the mean ± SD. Experiments were performed in triplicate. ** *p*<0.01, *** *p*<0.001, n.s. not significant.


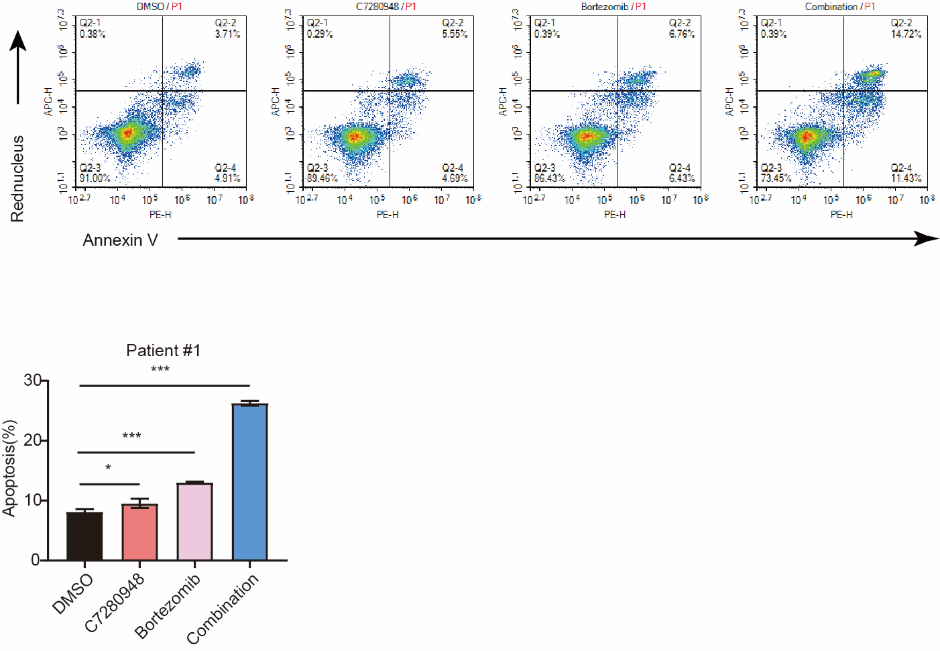


**Figure S7 Synergistic cytotoxic effect of combined PRMT1 inhibitor and BTZ in MM**

Apoptotic analysis of CD138^+^ cells isolated from NDMM patients treated for 48 h with C7280948 (200μM) and BTZ (5nM), alone or in combination by flow cytometry.

Data represent the mean ± SD. Experiments were performed in triplicate. * *p*<0.05, *** *p*<0.001.

Supplementary Methods

**Cell culture**

The human MM cell lines MM.1S, RPMI-8226, NCI-H929, U266, and OPM2 were cultured in RPMI-1640 (Hyclone, Logan, UT, USA) supplemented with 10% fetal bovine serum (FBS) (Gibco, Waltham, USA), 1% penicillin-streptomycin (100U/ml Thermo Fisher Scientific, Inc). The NCI-H929 cell media required the addition of β-mercaptoethanol to a final concentration of 0.05mM. 293T cells were maintained in DMEM (Hyclone, Logan, UT, USA) with 10% FBS and 1% penicillin-streptomycin. All cells were incubated at 37 °C in a 5% CO_2_ incubator. All cell lines were obtained from American Type Culture Collection (ATCC, Rockville, MD, USA) and were negative for mycoplasma contamination.

**Generation of PRMT1 knockout (KO) cells by LentiCrispr/CAS9**

According to the protocol previously documented by Zhang et al. ^19^, gRNAs were designed using the online CRISPR Design Tool (http://tools.genome-engineering.org). The PRMT1 KO guide RNA Sequences are shown in Supplementary Table 4. LentiCrisp v2 was used for cloning the following gRNAs into the expressing vector. Lentiviruses were made using LentiCripr-gRNA, packaging psPAX2 and pMD2.G in a 10:10:1 ratio in the six-well plate of HEK293T cells. Lentiviral supernatant was used to transduce cell lines in the six-well plates with 5μg/ml polybrene. MM.1S cells were transfected with Lentiviral supernatant and then selected with GFP+ by flow cytometry. Cells were then verified for knockout efficiency by Western blotting.

**Quantitative real-time polymerase chain reaction (****qRT-PCR)**

Total RNA was extracted from the bone marrow mononuclear cells (BMMC) using TRIzol® Reagent (Invitrogen, Germany). Then NanoDrop ND-1000 was used to determine the RNA purity and concentration. The cDNA was synthesized from total RNA using a Primescript RT reagent kit (Takara). Then, qRT-PCR was performed by SYBR Premix Ex Taq™ II (CWBIO, China). The reaction conditions were as follows: 95 °C for 30 s, 40 cycles of 95 °C for 5 s, and 60 °C for 30 s. The above experimental steps were operated in accordance with the manufacturer's protocols. Primers were synthesized by Tsingke (Beijing, China), and the sequences used are listed in Supplementary Table 5. The 2^-ΔΔCT^ method was used to calculate the gene expression level.

**Western blotting and Co-immunoprecipitation assay (Co-IP)**

Western blotting assay was conducted as described previously ^20^. Cell lysate samples were collected in RIPA buffer (POINEER Biotechnology, Xi'an, China), including a protease inhibitor cocktail (NCM Biotech, Suzhou, Xi'an). About 60μg protein lysates were separated with 10% SDS-polyacrylamide gels and transfected to PVDF membranes. Then the membranes were blocked into 5% BSA/PBST for 1h at room temperature and incubated with primary antibodies at 4°C overnight. Next, the membranes were incubated with secondary antibodies for 1h at room temperature. Finally, the protein bands were visualized with chemiluminescence reagents (ShareBio, Shanghai, China). The antibodies were used in this study as follows: PRMT1 (cat. no. 2449; Cell Signaling Technology, CST), NDUFS6 (cat. no. ab195807; Abcam), GAPDH (cat. no. 2118S; CST), METTL3 (cat. no. 86132; CST), METTL14 (cat. no. 51104; CST), WTAP (cat. no. 56501; CST), YTHDF2 (cat. no. 71283; CST), FLAG (cat. no. 14793; CST), Lamin B1 (cat. no. 9087; CST).

Co-IP was performed using the Pierce Crosslink Magnetic IP/Co-IP Kit (Thermo Fisher Scientific, China) according to the manufacturer's protocols. The antibody was first crosslinked with the magnetic beads. Then the cell lysate was incubated with the antibody and magnetic beads overnight at 4 ° C. Finally, the antigen was eluted. Target protein levels were detected by western blotting.

**Cell nucleus/cytoplasm isolation**

293T cells were cultured and washed with cold PBS. Nucleo-cytoplasmic fractionation was performed using a PARIS™ kit (Thermo Fisher Scientific, Inc.) according to the manufacturer's instructions. GAPDH and Lamin B1 were used as markers of cytoplasm and nucleus during western blotting.

**Transmission electron microscopy (TEM)**

MM.1S cells transfected with siRNA for 72h were collected and fixed with 2.5% glutaraldehyde fixative (containing 0.1M phosphate buffer and 4% paraformaldehyde) at 4°C for 2h. Next, cells were immersed in 0.1M phosphate buffer for 30 minutes, fixed in 1% osmium tetroxide fixative at 4℃ for 2 hours, and immersed in 0.1M phosphate buffer for 10 minutes. In addition, to prepare the MM cells for observation, they were subjected to ethanol gradient dehydration using the following steps: 30% ethanol for 10 minutes, 50% ethanol for 10 minutes, 70% ethanol for 10 minutes, overnight incubation in a solution of 70% ethanol, acetate uranium dioxy block dye; followed by two 10-minute washes in 90% ethanol and three 10-minute washes in 100% ethanol. Then, after replacement, impregnation, embedding, section, and staining with methylene blue, we observed samples under a JEM-100SX transmission electron microscope (HITACHI, Japan).

**RNA decay assay**

MM cells transfected with siRNA for 72h were treated with Actinomycin D (5μg/ml, MCE, USA) for 0, 6, and 8h. Then total RNA was isolated, and qRT-PCR was conducted as described previously. 18s rRNA was used as an internal control. The RNA lifetime (t_1/2_) was calculated using the equations below ^21^: Nt/N0 = e ^– Kdecay*t^, t_1/2_ = ln2/K_decay_.

**Methylated RNA immunoprecipitation sequencing (MeRIP-seq) and** **RNA sequencing (RNA-seq)**

Total RNA was isolated and purified as previously described. The RNA integrity was assessed by Bioanalyzer 2100 (Agilent, CA, USA). Dynabeads Oligo (dT)25-61005 (Thermo Fisher, CA, USA) was used to capture Poly (A) RNA. Then the Poly (A) RNA was incised into pieces under 86 ° C for 7 minutes by Magnesium RNA Fragmentation Module (NEB, cat. e6150, USA). In the IP buffer (50 mM Tris-HCl, 750 mM NaCl and 0.5% Igepal CA-630), the cleaved RNA fragments and m^6^A Antibody (No. 202003, Synaptic Systems, Germany) were premixed and IP. Then DNA was synthesized, and the compound duplex of DNA and RNA was converted into DNA duplex. Next, dUTP (Thermo Fisher, article No. R0133, USA) was incorporated into the duplex. An A base was added to the end of the double-stranded DNA, and the fragment size was screened and purified using magnetic beads (insert size: 180-220bp). UDG enzyme (NEB, cat.m0280, USA) was used to digest the two strands, and then PCR was performed under the following conditions to form a sequencing library: initial denaturation at 95℃ for 3 min; 8 cycles of denaturation at 98℃ for 15 sec, annealing at 60℃ for 15 sec, and extension at 72℃ for 30 sec; and then final extension at 72℃ for 5 min. At last, Illumina Novaseq™ 6000 (LC-Bio Technology CO., Ltd., Hangzhou, China) was used for double-ended sequencing with PE150 sequencing mode following the standard protocol.

For RNA sequencing, total RNA was isolated with TRIzol regent following the protocol as described previously. Other processes were performed by Omic Studio technologies (Hangzhou, China).

**RNA immunoprecipitation (RIP) assay**

RIP-qPCR was performed using the BersinBioTM RNA Immunoprecipitation Kit (BersinBio, Guangzhou, China) according to the manufacturer’s instruction. Cell lysates were divided into 3 groups, anti-IgG, anti-YTHDF2, and input. Then Polysome lysis buffer was used to lyse cells and the cell lysate was divided into anti-YTHDF2, anti-IgG (1mg/ml, Cell Signaling Technology) and input samples. Then, 5μg specific antibodies of anti-IgG or anti-YTHDF2, protein A/G magnetic beads and cell lysates were incubated at 4 ℃ overnight. The lysates were washed, then digested with Proteinase K for 1 h at 55 ° C. At last, the RNA was purified with mixture of phenol: chloroform: Isoamyl Alcohol (25:24:1). Target RNA levels were detected by qRT-PCR.

**MeRIP-qPCR**

MeRIP assay was performed by the BersinBioTM MeRIP Kit (BersinBio, Guangzhou, China) according to the manufacturer’s instruction. Firstly, we used TRIzol regent to isolate total RNA as described previously. Then the RNA was segmented into about 300-nucleotide fragments. Next, the fragments were incubated with 5μg anti-IgG or anti-m^6^A (68055-1-Ig, Proteintech) for 2 h at 4 ℃. Moreover, we mixed Protein A/G magnetic beads and antibody-treated RNA in IP buffer for 2 h at 4 ℃. The RNA was washed, then eluted with Proteinase K and elution buffer for 1 h at 55 ° C. At last, the RNA was purified with mixture of phenol: chloroform: Isoamyl Alcohol (25:24:1). The methylated RNA expression was measured by qRT-PCR.

**Immunohistochemistry and histological analysis**

The paraffin-embedded sections (4μm-thick) from xenograft mice tumors were incubated with antibodies directed against ki-67 (cat. no. 12202; 1:500 dilution, CST), PRMT1 (cat. no. ab190892; 1:500 dilution, Abcam), NDUFS6 (cat. no. ab195807; 1:500 dilution, Abcam) overnight at 4℃. Then, the sections were incubated with specific secondary antibodies for 1 h at room temperature and treated with DAB. Moreover, the paraffin-embedded sections were stained with hematoxylin-eosin (HE) to evaluate histological changes.

**References**

1. Genome engineering using the CRISPR-Cas9 system. *Nature protocols* 2013.

2. Shen Y, Feng YD, Li FM, Jia YC, Peng Y, Zhao WH*, et al.* lncRNA ST3GAL6-AS1 promotes invasion by inhibiting hnRNPA2B1-mediated ST3GAL6 expression in multiple myeloma. *International Journal of Oncology* 2021, **58**(4).

3. Fang R, Chen X, Zhang S, Shi H, Ye Y, Shi H*, et al.* EGFR/SRC/ERK-stabilized YTHDF2 promotes cholesterol dysregulation and invasive growth of glioblastoma. *Nature communications* 2021, **12**(1)**:** 177.

Supplementary Table

Supplementary Table 1 Clinical characteristics of 31 MM patients

| Clinical characteristics | Number of cases | proportion（%） |
| --- | --- | --- |
| **Gender** | | |
| male | 16 | 52% |
| female | 15 | 48% |
| **Age** |  |  |
| <65 | 22 | 71% |
| ≥65 | 9 | 29% |
| **Type** |  |  |
| IgA | 5 | 16% |
| IgG | 10 | 32% |
| IgD | 4 | 13% |
| Light chain | 9 | 29% |
| Non-secretory | 3 | 10% |
| **DSS stage** | | |
| II | 2 | 6% |
| III | 29 | 94% |
| **ISS stage** |  |  |
| I | 3 | 10% |
| II | 6 | 19% |
| III | 22 | 71% |
| **R-ISS stage** |  |  |
| I | 3 | 10% |
| II | 13 | 42% |
| III | 15 | 48% |
| **mSMART** |  |  |
| Standard risk | 5 | 16% |
| High risk | 22 | 71% |
| unknown | 4 | 13% |
| **Cytogenetic abnormalitics** |  |  |
| del 13q14 | 8 | 26% |
| del RB1 | 14 | 45% |
| IgH rearrangement | 14 | 45% |
| 1q21 gain or amplication | 17 | 55% |
| t4, 14 | 2 | 6% |
| t11, 14 | 2 | 6% |
| t14,16 | 1 | 3% |
| none | 6 | 19% |

Supplementary Table 2. siRNA sequences of genes used in this study.

| gene | Target sequence |
| --- | --- |
| si-PRMT1-1 | GCAACTCCATGTTTCATAA |
| si-PRMT1-2 | AGACGGTGTTCTACATGGA |
| si-PRMT1-3 | CCATCGACCTGGACTTCAA |
| si-NDUFS6-1 | GTTTGTAGGTCGTCAGAAA |
| si-NDUFS6-2 | CTTTGCCATTGATTTGATA |
| si-NDUFS6-3 | CCACCCAAAAGTGTATATA |
| si-WTAP | GAAGCATATGTACAAGCTT |
| si-METTL3 | GGACTCGACTACAGTAGCT |
| si-METTL14 | GGACCAACGCTTACAAATA |
| si-YTHDF2 | GCACAGAAGTTGCAAGCAA |

Supplementary Table 3. Univariate analysis and Multivariate analysis for OS in GSE136324

| variables | univariate analysis | | multivariate analysis | | | | |
| --- | --- | --- | --- | --- | --- | --- | --- |
|  | HR (95%CI) | *p* value | beta | SE | Wald | HR (95%CI) | *p* value |
| Age,years | 1.045(1.031-1.060) | **<0.0001** | 0.045 | 0.007 | 40.978 | 1.046（1.032-1.061） | **<0.0001** |
| Gender | 1.177(0.944-1.468) | 0.147 |  |  |  |  |  |
| LDH(U/l) | 1.003(1.001-1.004) | **<0.0001** | 0.002 | 0.001 | 8.816 | 1.002(1.001-1.003) | **0.003** |
| ALB(g/l) | 0.654(0.566-0.756) | **<0.0001** | -0.293 | 0.076 | 14.724 | 0.746(0.642-0.866) | **<0.0001** |
| ISS stage | 1.615(1.346-1.938) | **<0.0001** | 0.304 | 0.100 | 9.183 | 1.355(1.113-1.650) | **0.002** |
| PRMT1 | 1.846(1.313-2.596) | **<0.0001** | 0.676 | 0.173 | 15.255 | 1.966(1.400-2.759) | **<0.0001** |

Supplementary Table 4 sgRNA for CRISPRi

| gene | Target sequence |
| --- | --- |
| Sg-PRMT1-1 | CGAGGCCGCGAACTGCATCA |
| Sg-PRMT1-2 | GCTCATCCCATTAGCCAAGG |
| Sg-PRMT1-3-1 | GTGGATGCCAAAGTGTGCGT |
| Sg-PRMT1-3-2 | GGATGTCATGTCCTCAGCGT |

Supplementary Table 5. Primers for qRT-PCR used in this study.

| **gene** | **Forward primer** | **Reverse primer** |
| --- | --- | --- |
| PRMT1 | TGCGGTGAAGATCGTCAAAGCC | GGACTCGTAGAAGAGGCAGTAG |
| 18s rRNA | CAGCCACCCGAGATTGAGCA | TAGTAGCGACGGGCGGTGTG |
| GAPDH | CGGAGTCAACGGATTTGGTCGTAT | AGCCTTCTCCATGGTGGTGAAGAC |
| COX6B1 | CTACAAGACCGCCCCTTTTGA | GCAGAGGGACTGGTACACAC |
| ATP6V1F | CTCATCGCAGTGATCGGAGAC | CGGTTCTTGTTAAGCTCCCCTAT |
| UQCRC1 | GGGGCACAAGTGCTATTGC | GTTGTCCAGCAGGCTAACC |
| ATP5ME | GAGCCACGCGCTACAATTAC | GCCAATTCTCTGGCAATCCG |
| UQCRQ | CGCGAGTTTGGGAATCTGAC | TAGTGAAGACGTGCGGATAGG |
| SDHD | CATCTCTCCACTGGACTAGCG | TCCATCGCAGAGCAAGGATTC |
| COX4I1 | CAGGGTATTTAGCCTAGTTGGC | GCCGATCCATATAAGCTGGGA |
| UQCRH | GAGGACGAGCAAAAGATGCTT | CGAGAGGAATCACGCTCATCA |
| UQCR10 | CCATCATCGTGGGCGTCATGTT | GCCTCCAAGGAACTACTTGTTCT |
| COX5A | ATCCAGTCAGTTCGCTGCTAT | CCAGGCATCTATATCTGGCTTG |
| NDUFS6 | TGGAGACTCGGGTGATAGCGTG | GTGGTGCTGTCTGAACTGGAGC |
| NDUFA4L2 | CTGGGACAGAAAGAACAACCCG | CAGCCTGGCTTAGAAGTCTGGC |
| NDUFA11 | TCACACTCAATCCTCCGGGCACCTT | TGATGCAGGTGGTGAGGCCAAACA |
| ATP5MC1 | CTGTTGTACCAGGGGTCTAATCA | GTGGGAAGTTGCTGTAGGAAG |
| NDUFB2 | AGCGGACTCATGTGGTTCTGGA | AGAGTGAGGCTGAGTCTACACC |
| COX8A | TGTACTCCGTGCCATCATGT | GGTCACGAAGCAGGAGGTAA |
| COX5B | GGAGATCATGCTGGCTGCAAAG | GCAGCCTACTATTCTCTTGTTGG |
| COX6A1 | TCTCACCTTCTTCGTCGCGCTC | GCTTGGTCCTGATGCGGAGATG |
