# Supplementary material for: PRMT1 methylation of WTAP promotes multiple myeloma tumorigenesis by activating oxidative phosphorylation via m6A modification of NDUFS6
Source: Cell Death Dis. 2023 Aug 9;14(8):512. doi: 10.1038/s41419-023-06036-z (PMC10412649; doi:10.1038/s41419-023-06036-z)
Supplement: Supplementary file 2 — Original Data File [file 41419_2023_6036_MOESM2_ESM.pdf]

Figure 1

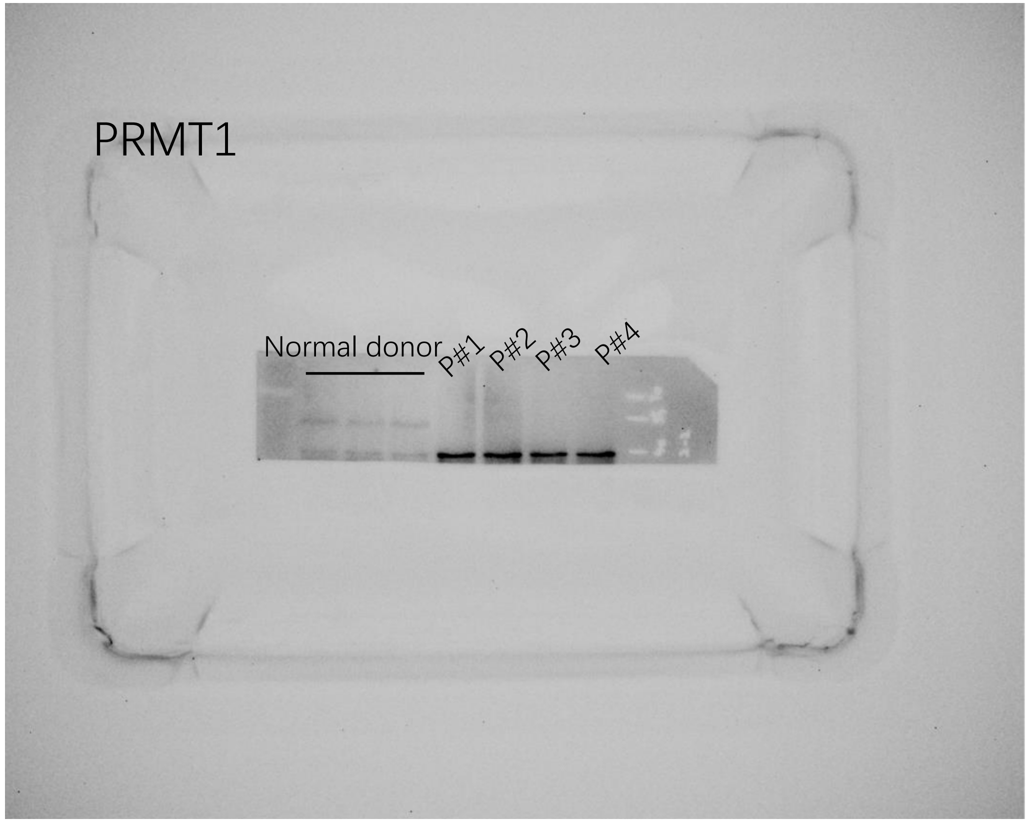

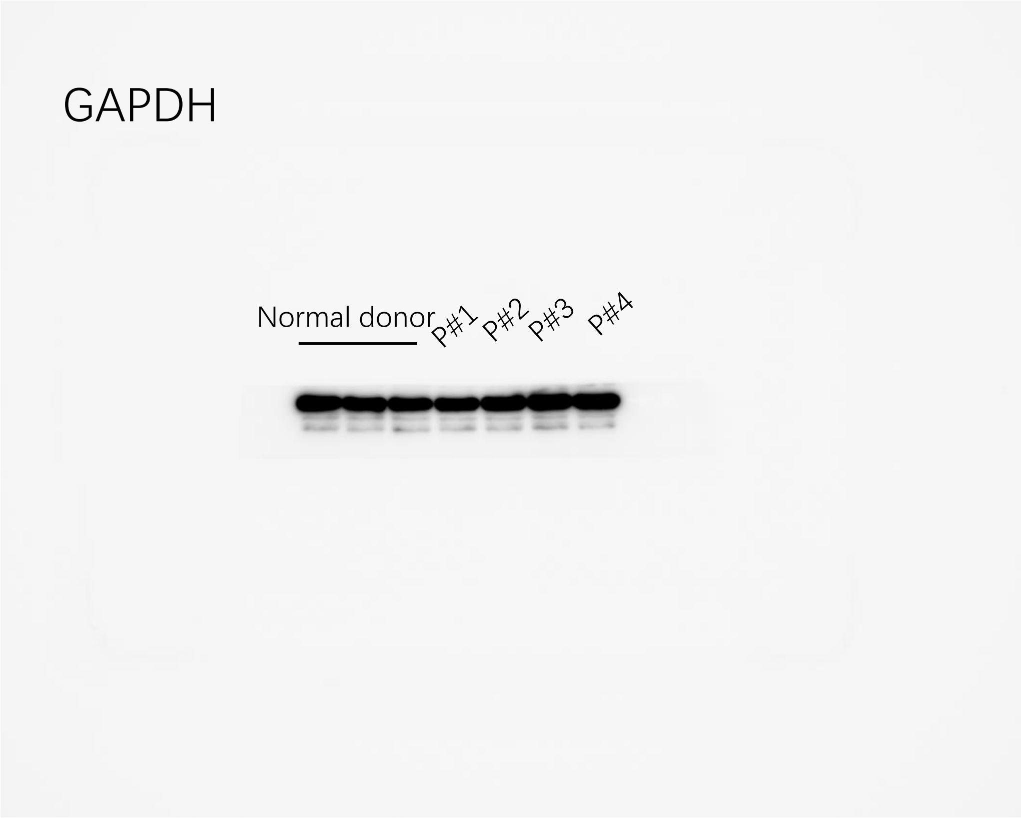

Figure 2

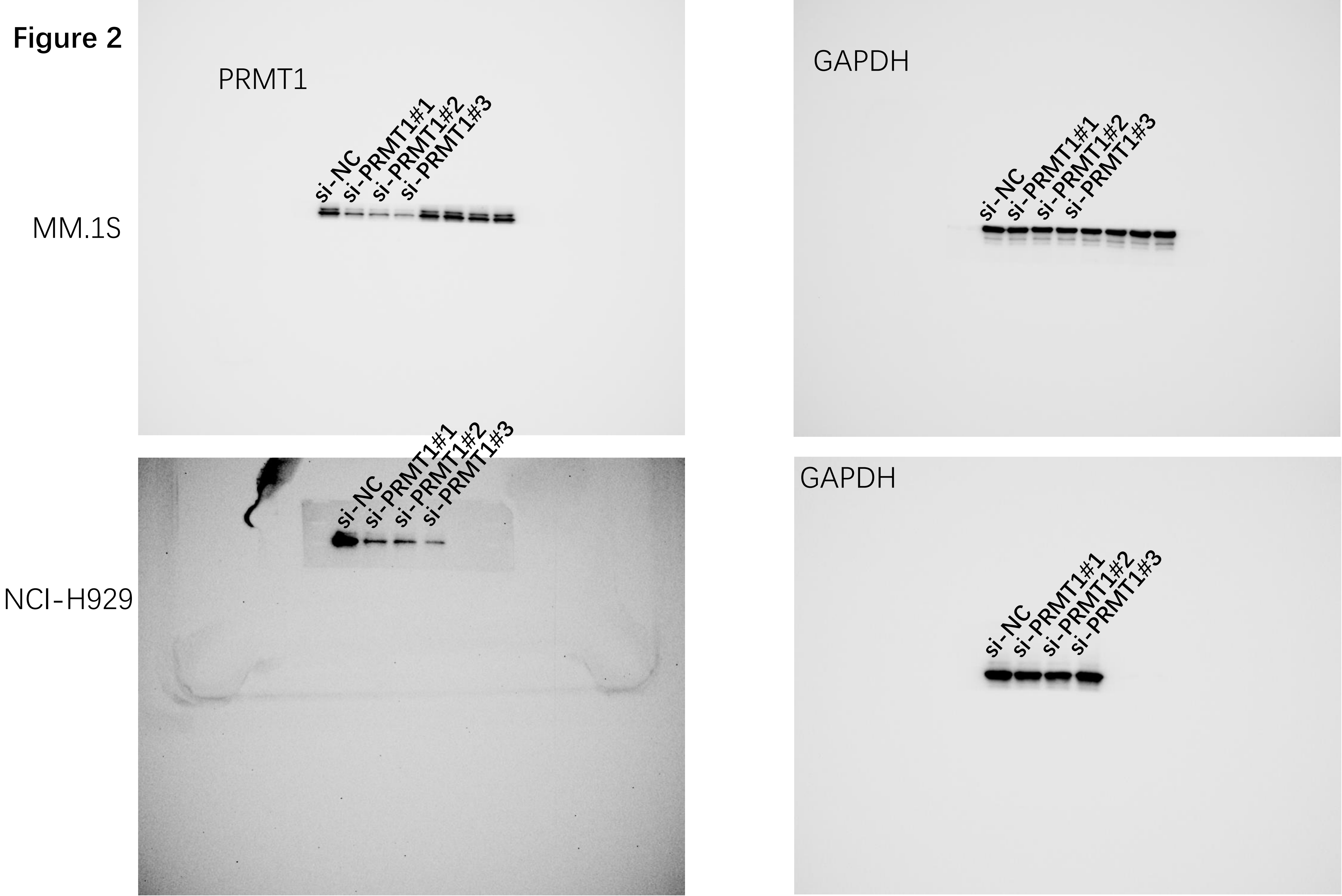
MM.1S
NCI-H929
PRMT1
GAPDH
si-NC
si-PRMT1#1
si-PRMT1#2
si-PRMT1#3
GAPDH
si-NC
si-PRMT1#1
si-PRMT1#2
si-PRMT1#3
si-NC
si-PRMT1#1
si-PRMT1#2
si-PRMT1#3
si-NC
si-PRMT1#1
si-PRMT1#2
si-PRMT1#3

Figure 4

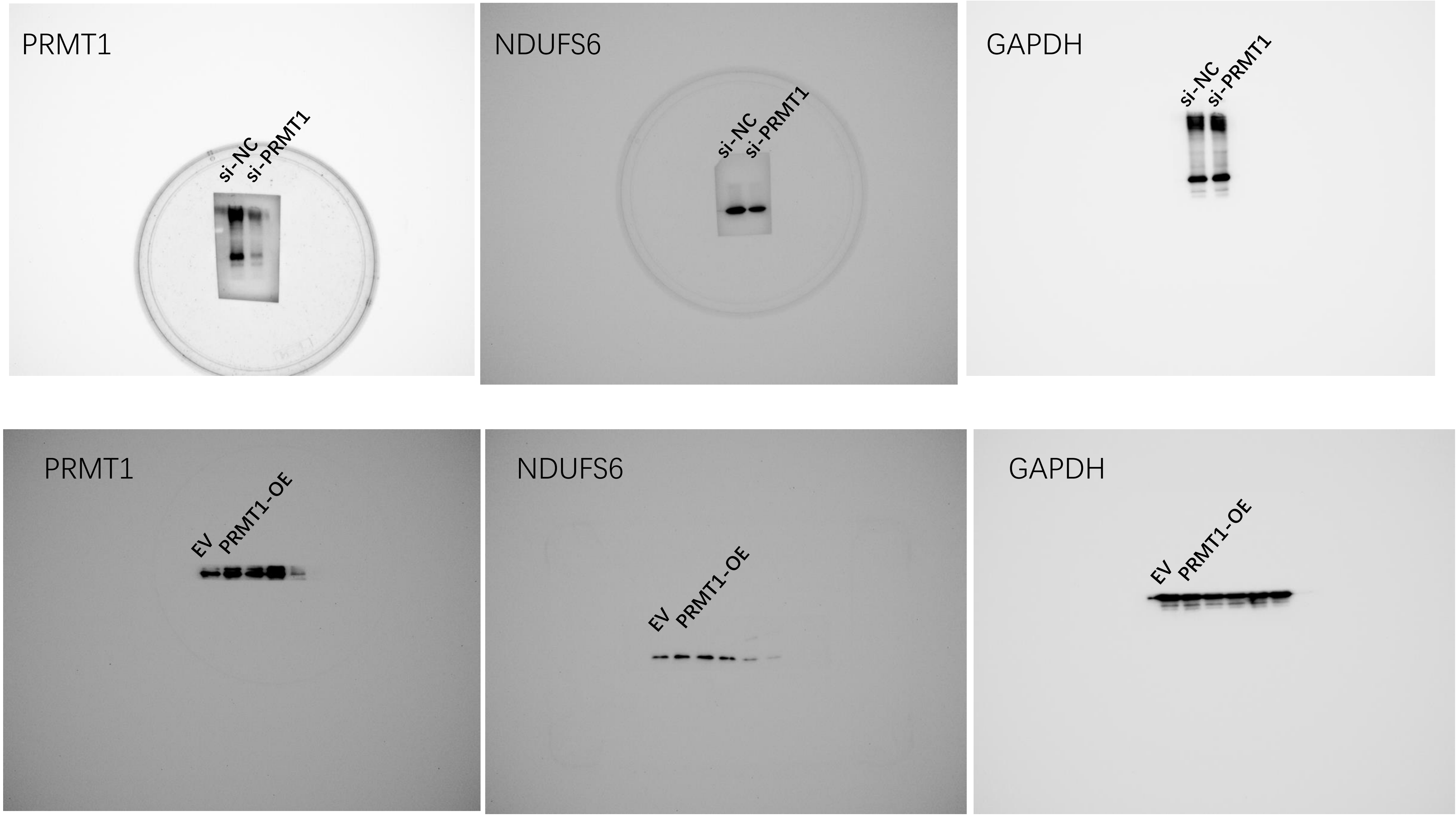

PRMT1
si-NC
si-PRMT1
NDUFS6
si-NC
si-PRMT1
GAPDH
si-NC
si-PRMT1
PRMT1
EV
PRMT1-OE
NDUFS6
EV
PRMT1-OE
GAPDH
EV
PRMT1-OE

Figure 4

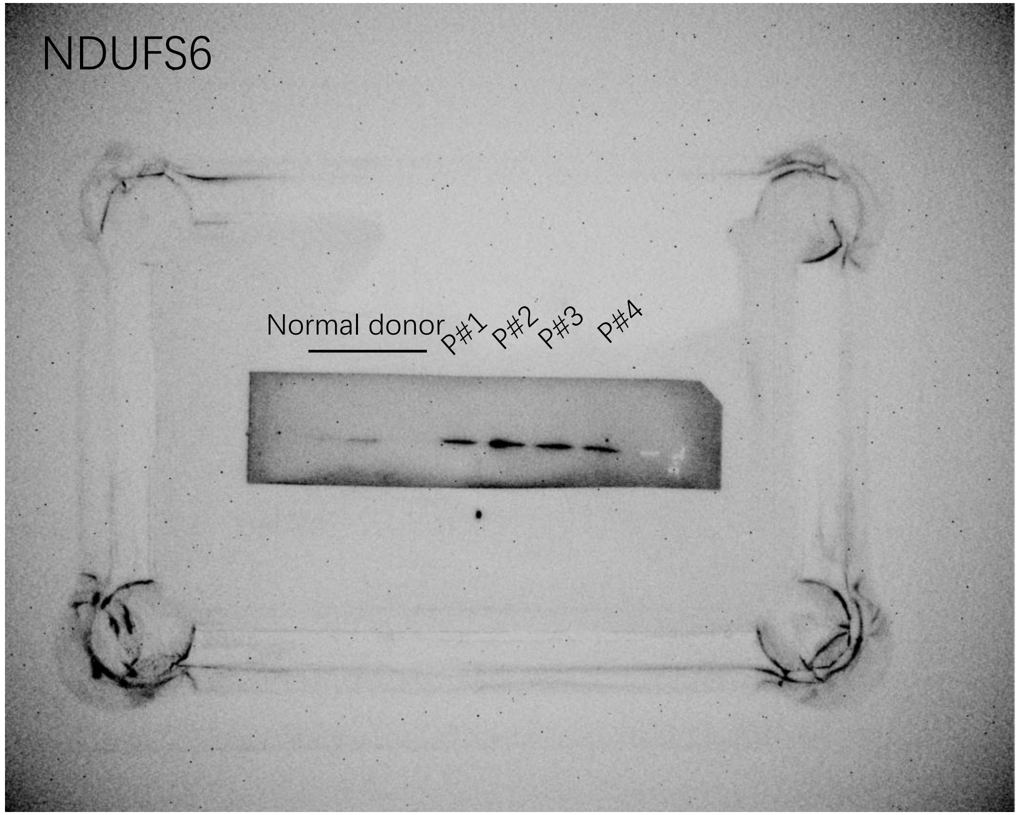

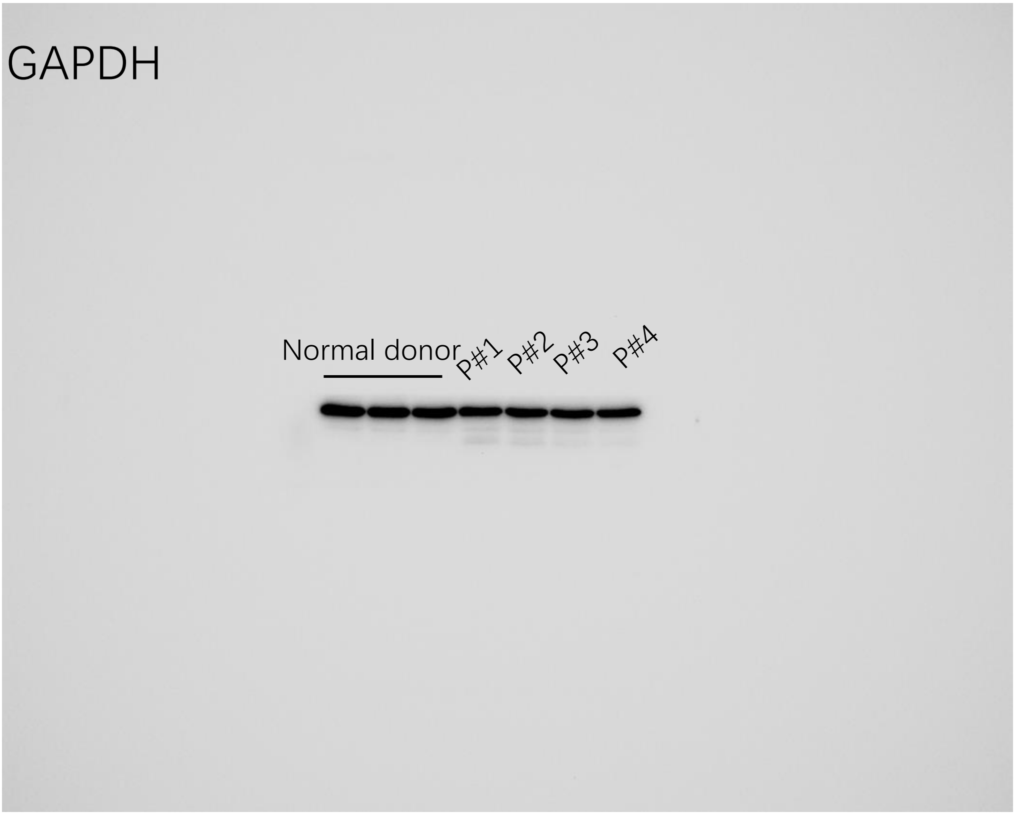

Figure 5

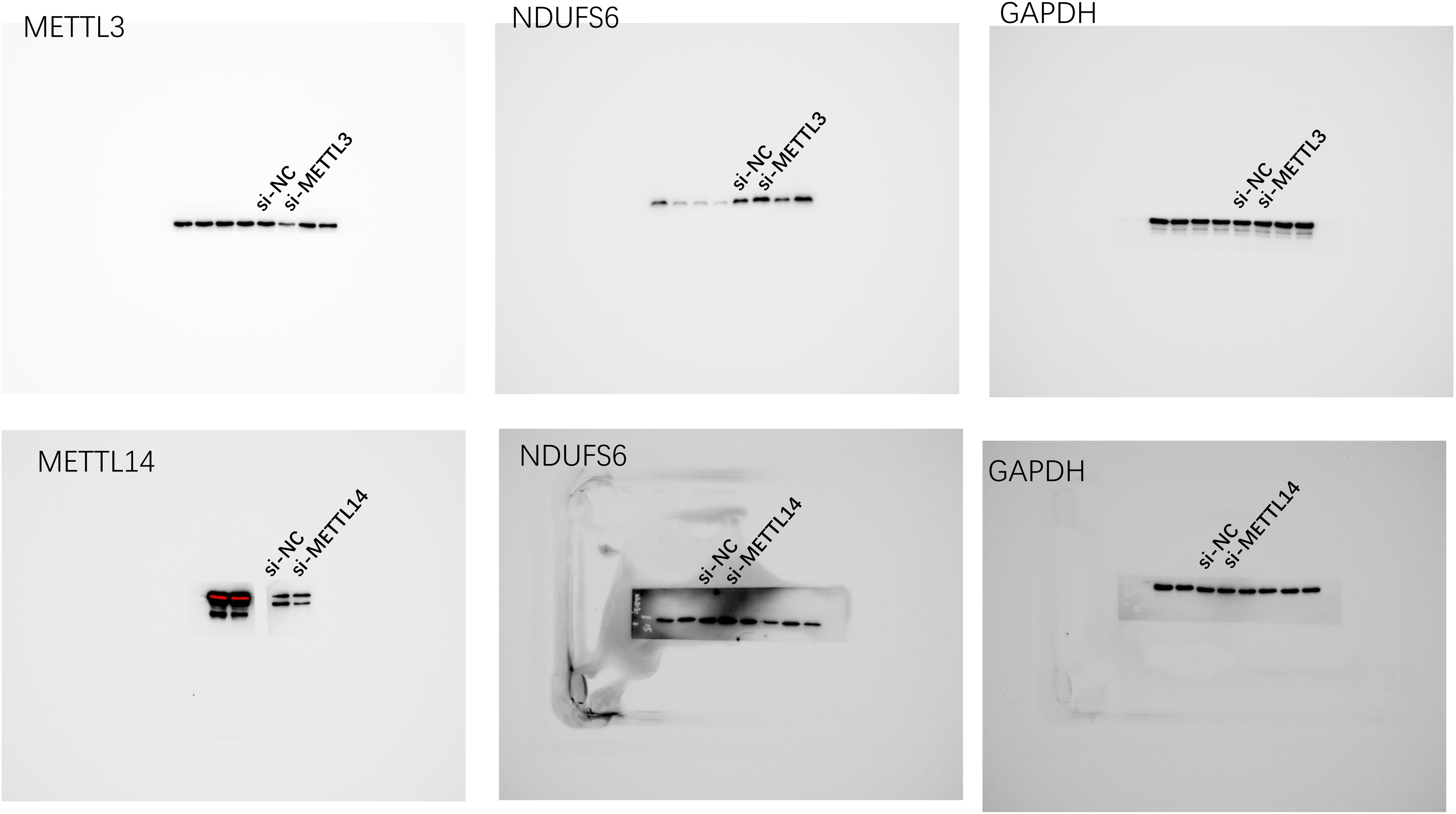
METTL3
si-NC
si-METTL3
NDUFS6
si-NC
si-METTL3
GAPDH
si-NC
si-METTL3
METTL14
si-NC
si-METTL14
NDUFS6
si-NC
si-METTL14
GAPDH
si-NC
si-METTL14

Figure 5

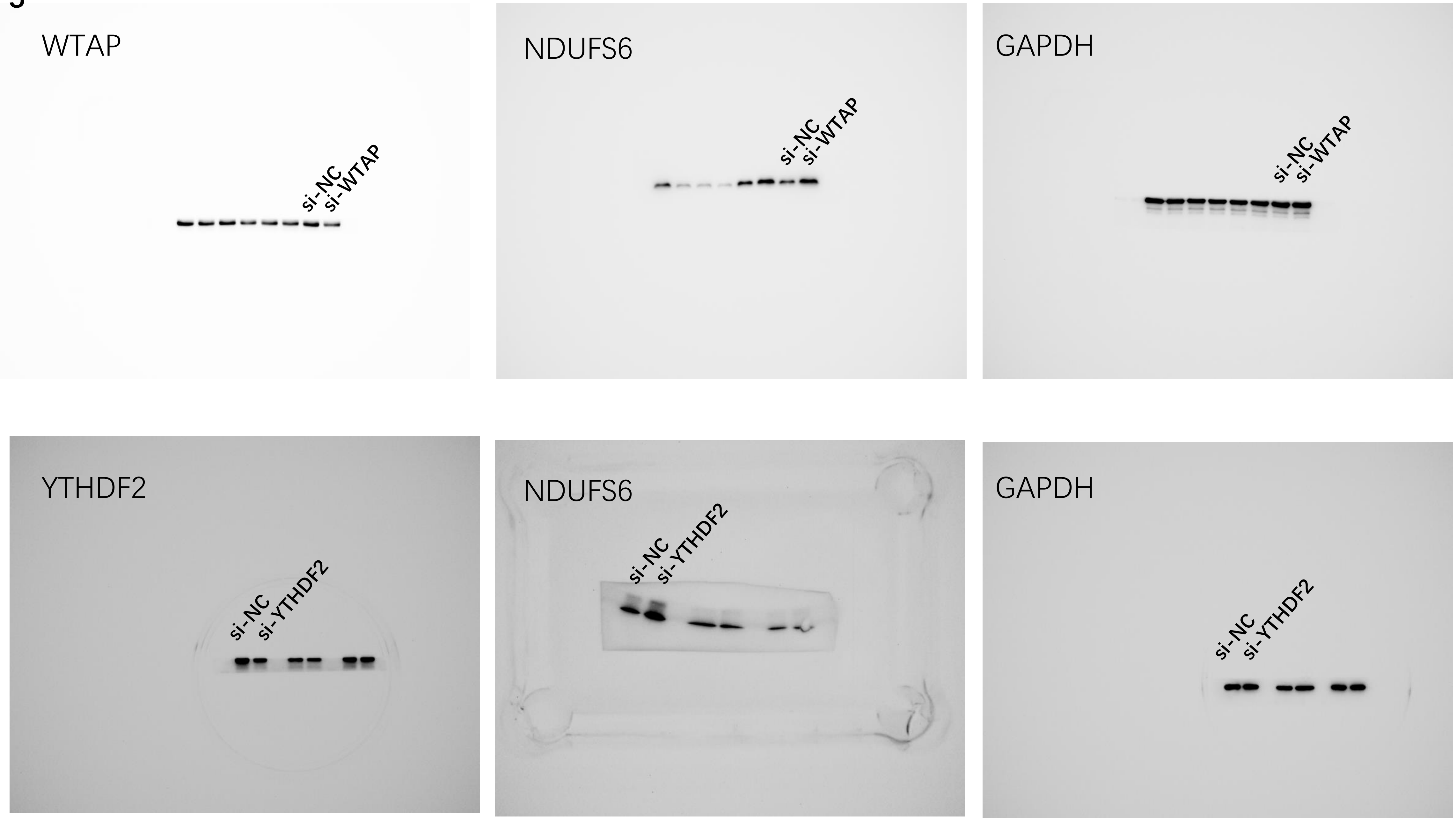
WTAP
si-NC
si-WTAP
NDUFS6
si-NC
si-WTAP
GAPDH
si-NC
si-WTAP
YTHDF2
si-NC
si-YTHDF2
NDUFS6
si-NC
si-YTHDF2
GAPDH
si-NC
si-YTHDF2

Figure 5

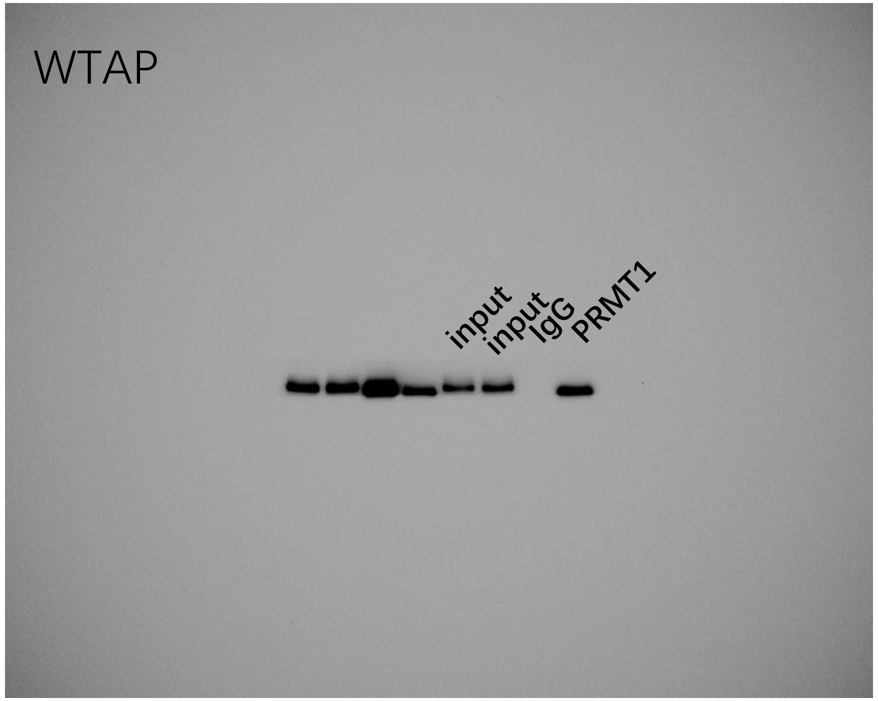

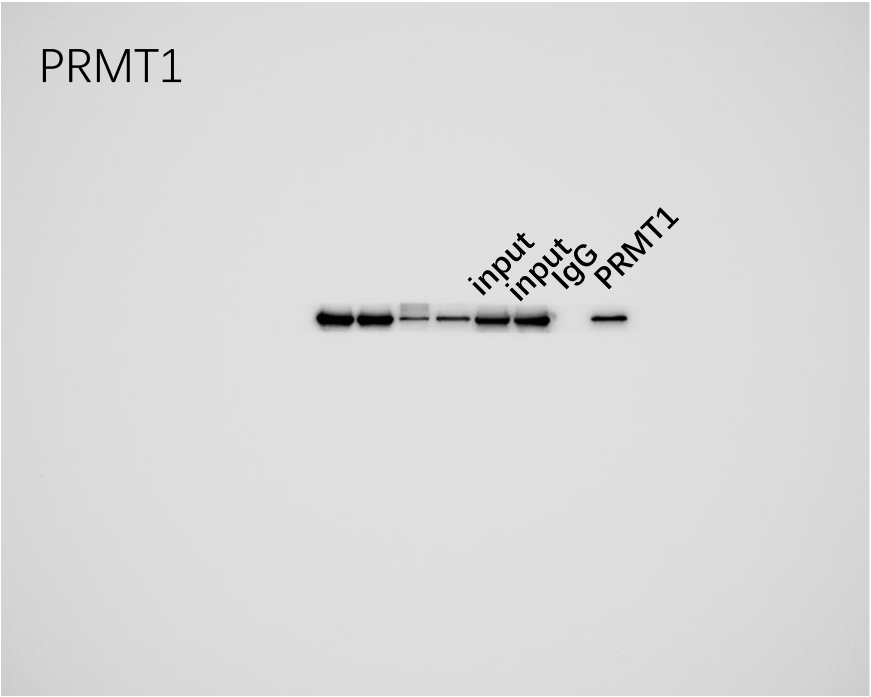

Figure 6

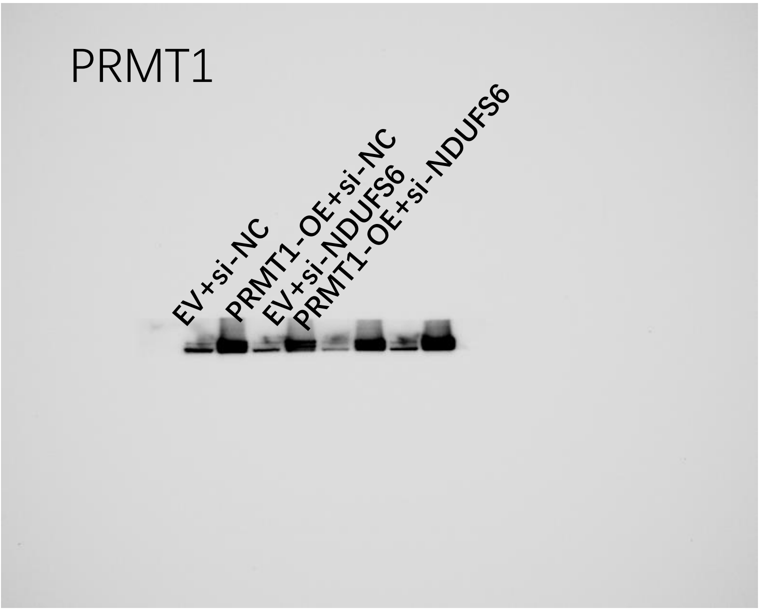
PRMT1
EV+si-NC
PRMT1-OE+si-NC
EV+si-NDUFS6
PRMT1-OE+si-NDUFS6

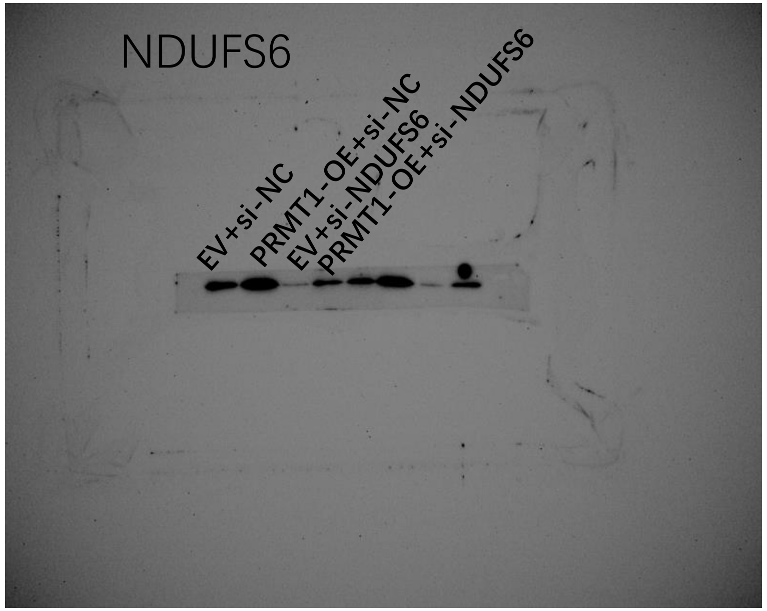
NDUFS6
EV+si-NC
PRMT1-OE+si-NC
EV+si-NDUFS6
PRMT1-OE+si-NDUFS6

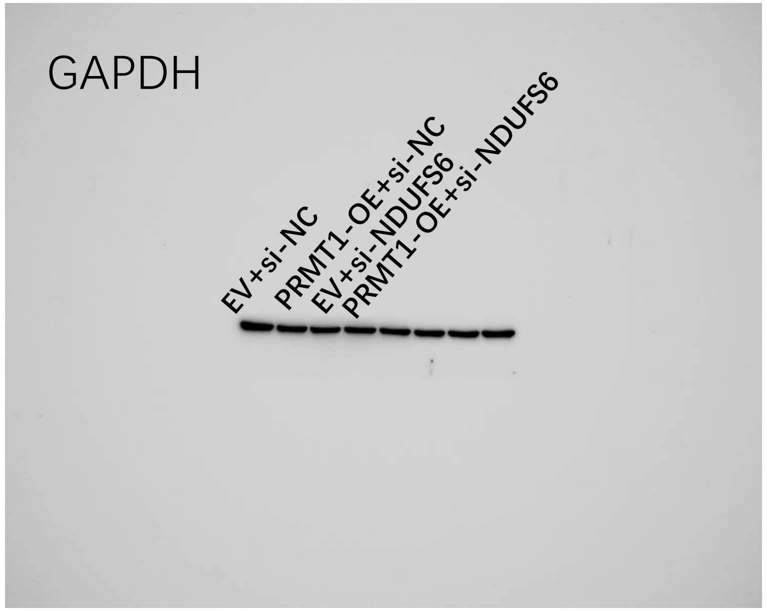
GAPDH
EV+si-NC
PRMT1-OE+si-NC
EV+si-NDUFS6
PRMT1-OE+si-NDUFS6

Figure 6

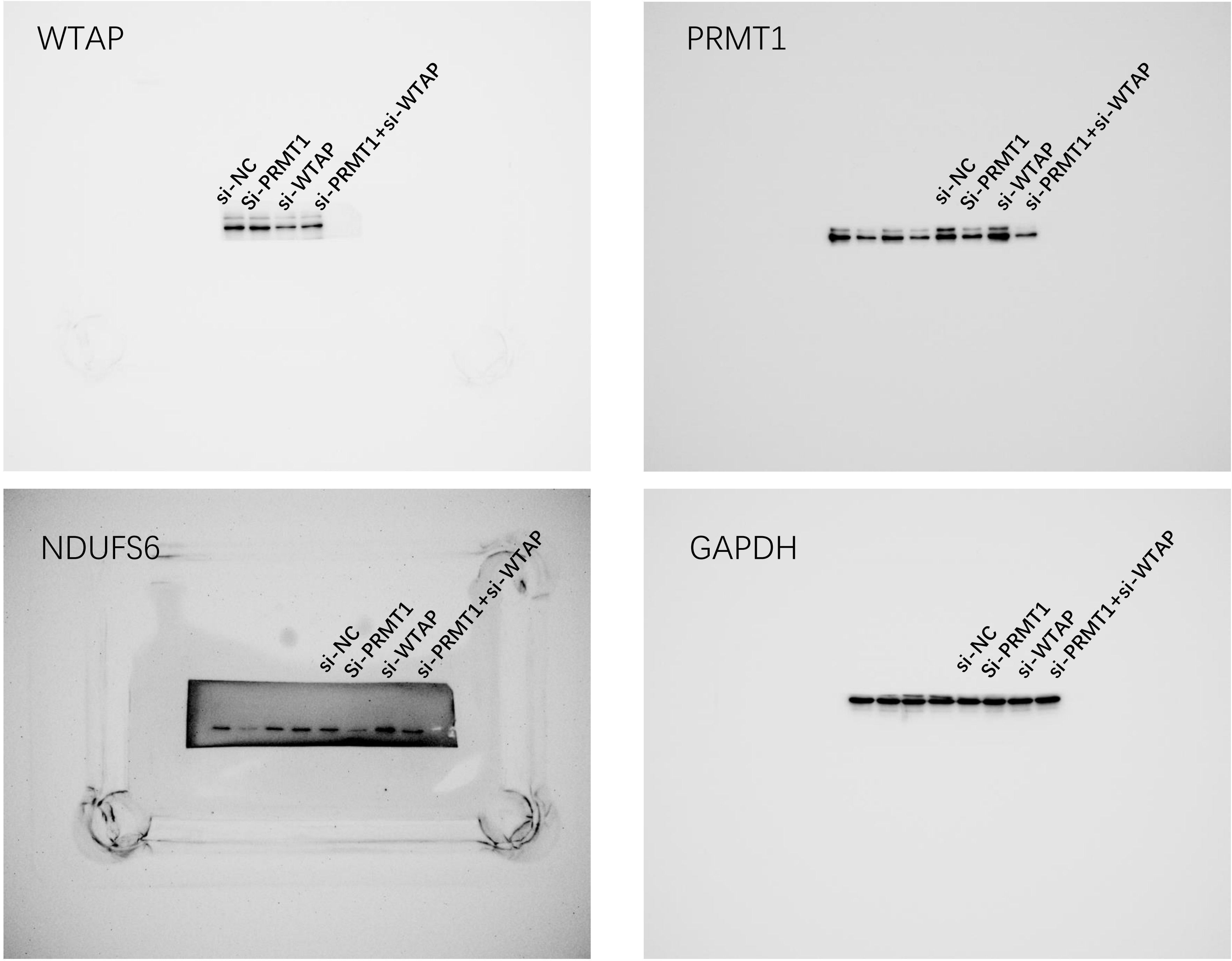
WTAP
si-NC
Si-PRMT1
si-WTAP
si-PRMT1+si-WTAP
PRMT1
si-NC
Si-PRMT1
si-WTAP
si-PRMT1+si-WTAP
NDUFS6
si-NC
Si-PRMT1
si-WTAP
si-PRMT1+si-WTAP
GAPDH
si-NC
Si-PRMT1
si-WTAP
si-PRMT1+si-WTAP

Figure S2

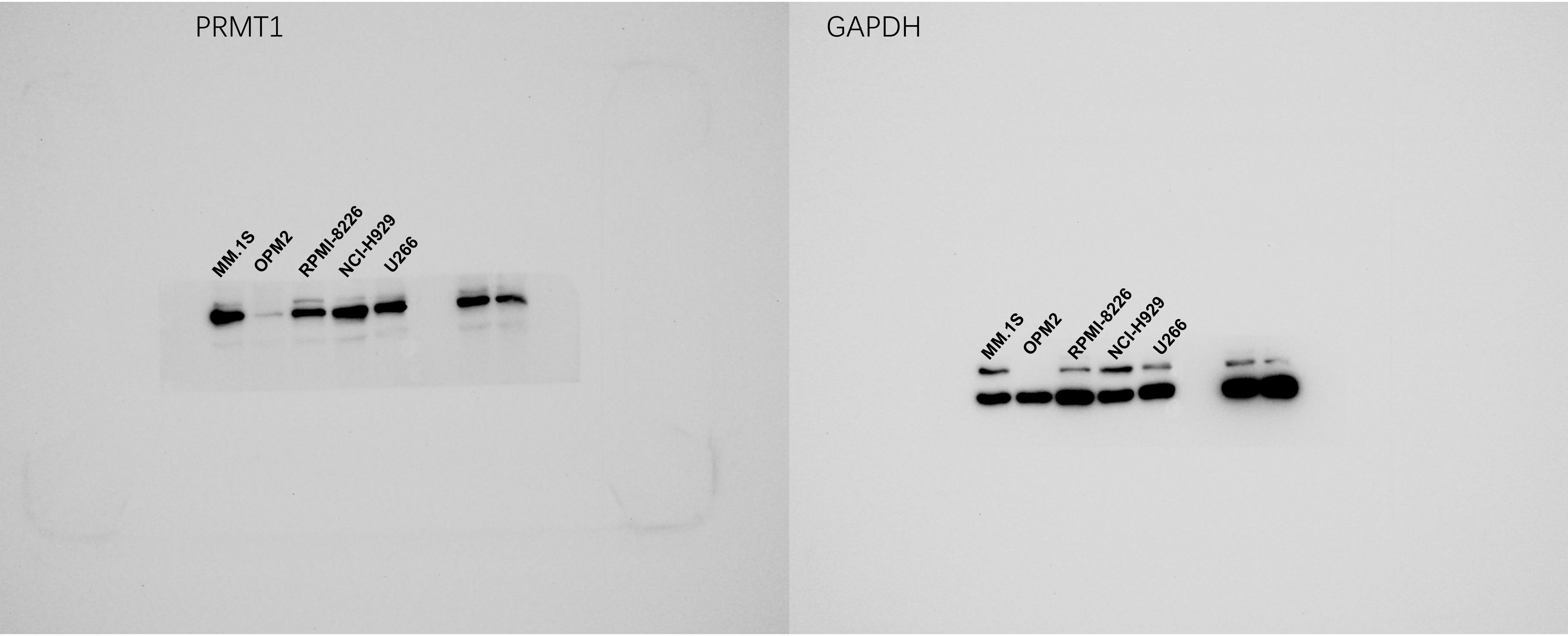
PRMT1
MM.1S
OPM2
RPMI-8226
NCI-H929
U266
GAPDH
MM.1S
OPM2
RPMI-8226
NCI-H929
U266

Figure S2

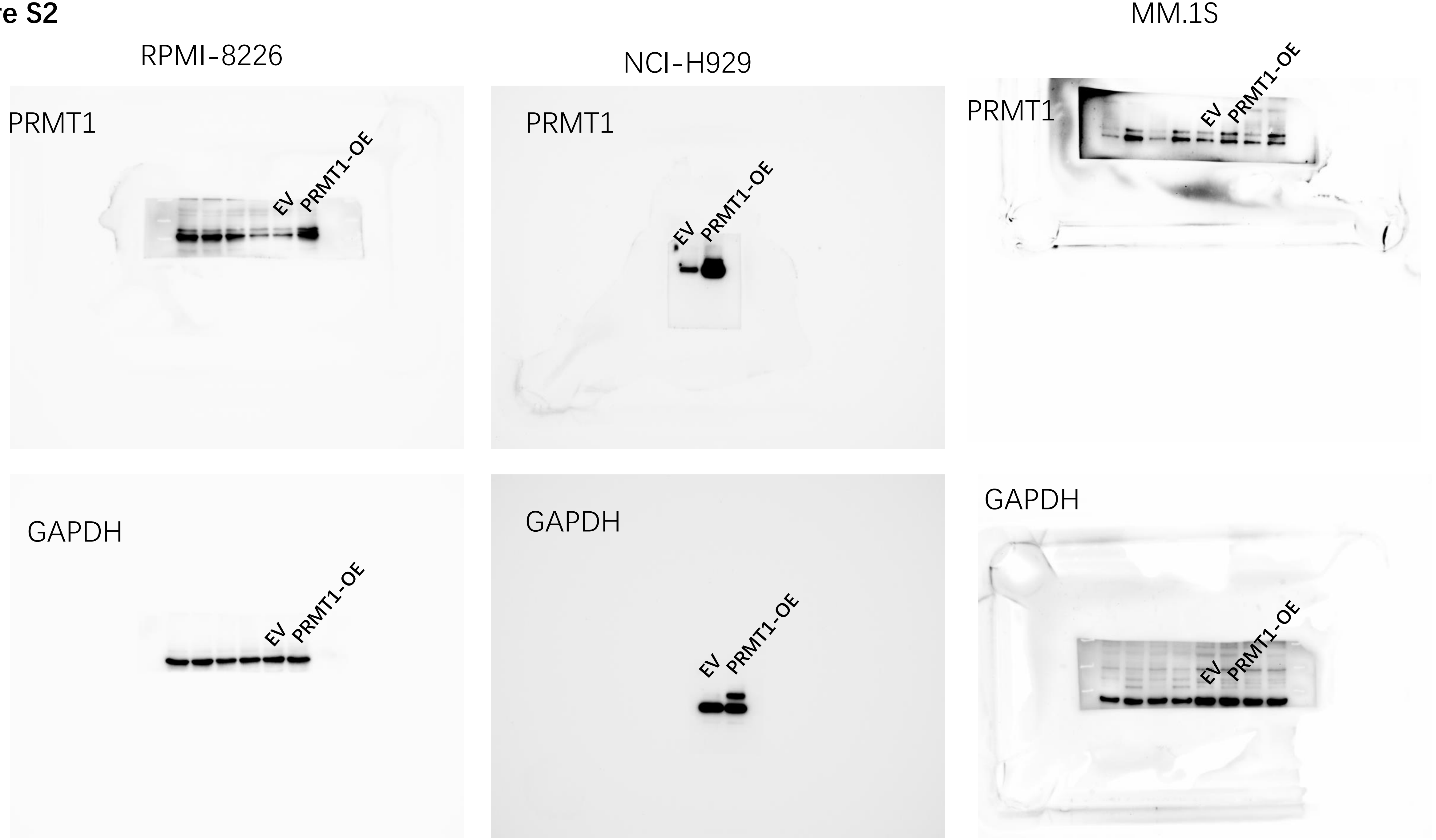
RPMI-8226
NCI-H929
MM.1S
PRMT1
EV
PRMT1-OE
PRMT1
EV
PRMT1-OE
PRMT1
EV
PRMT1-OE
GAPDH
EV
PRMT1-OE
GAPDH
EV
PRMT1-OE
GAPDH
EV
PRMT1-OE

Figure S3

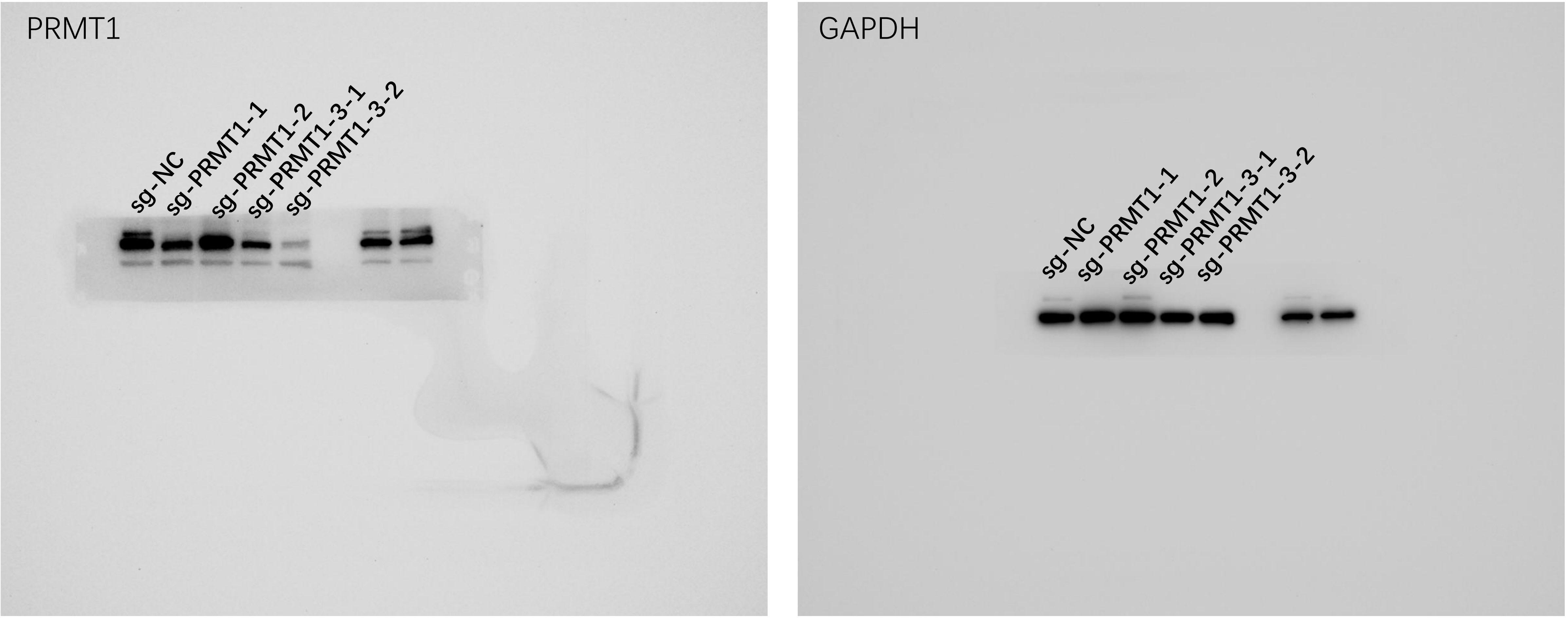
PRMT1
sg-NC
sg-PRMT1-1
sg-PRMT1-2
sg-PRMT1-3-1
sg-PRMT1-3-2
GAPDH
sg-NC
sg-PRMT1-1
sg-PRMT1-2
sg-PRMT1-3-1
sg-PRMT1-3-2

Figure S5

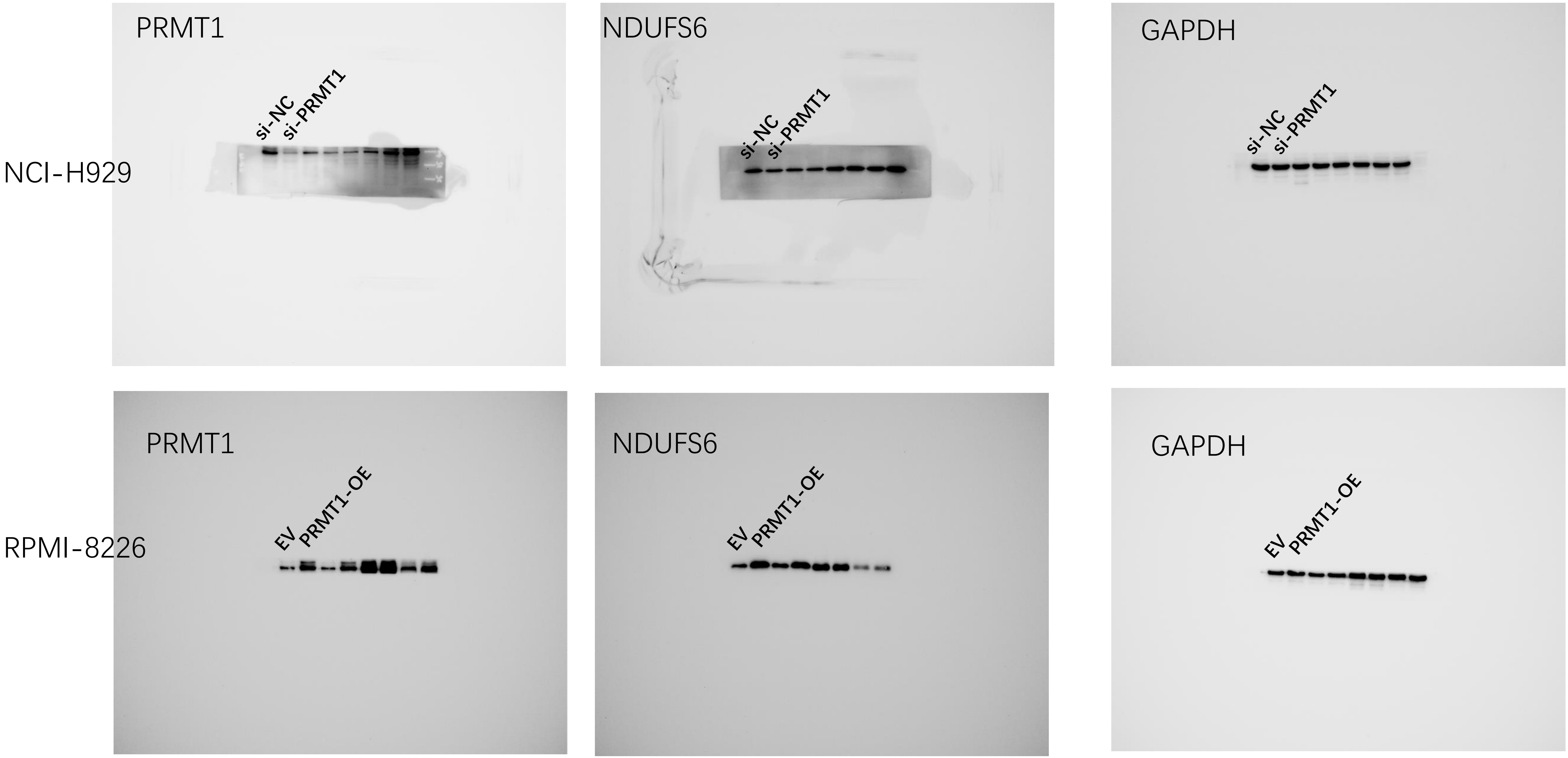
PRMT1
NDUFS6
GAPDH
NCI-H929
si-NC
si-PRMT1
RPMI-8226
EV
PRMT1-OE

Figure S5

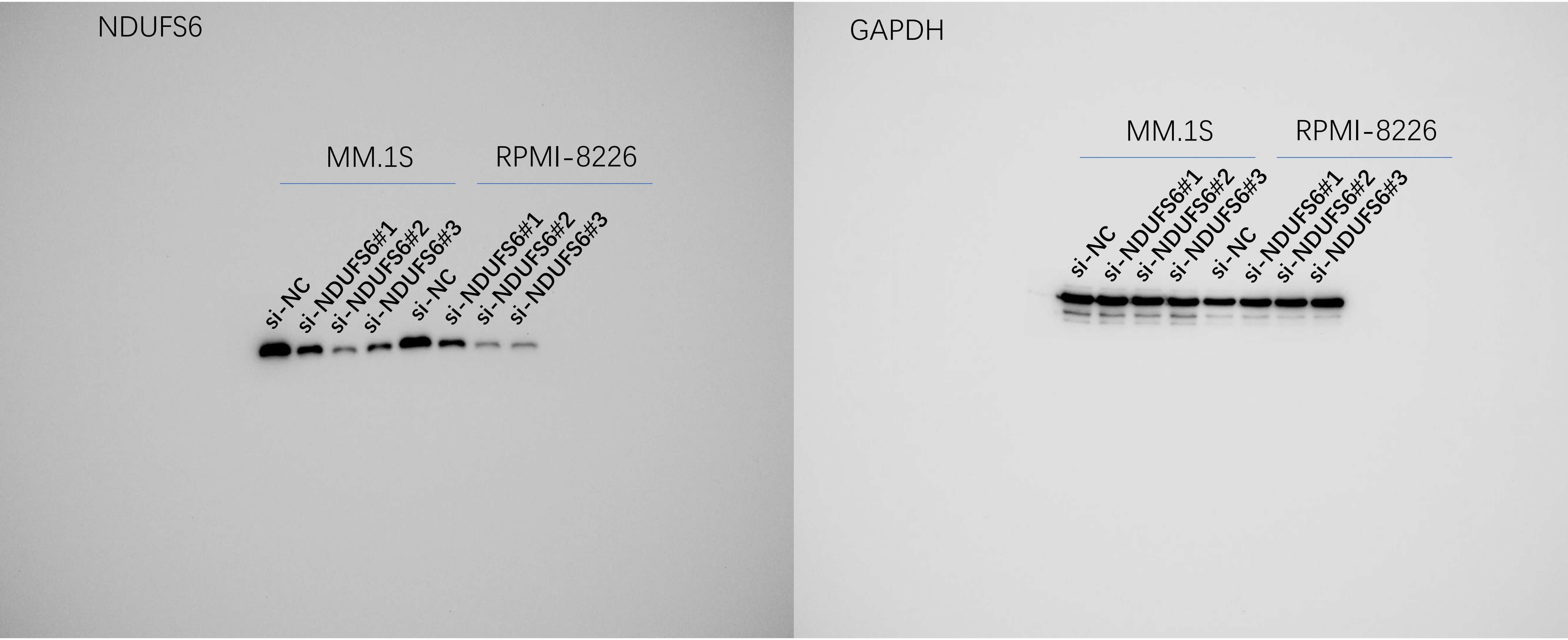

NDUFS6
MM.1S
RPMI-8226
si-NC
si-NDUFS6#1
si-NDUFS6#2
si-NDUFS6#3
si-NC
si-NDUFS6#1
si-NDUFS6#2
si-NDUFS6#3
GAPDH
MM.1S
RPMI-8226
si-NC
si-NDUFS6#1
si-NDUFS6#2
si-NDUFS6#3
si-NC
si-NDUFS6#1
si-NDUFS6#2
si-NDUFS6#3

Figure S6

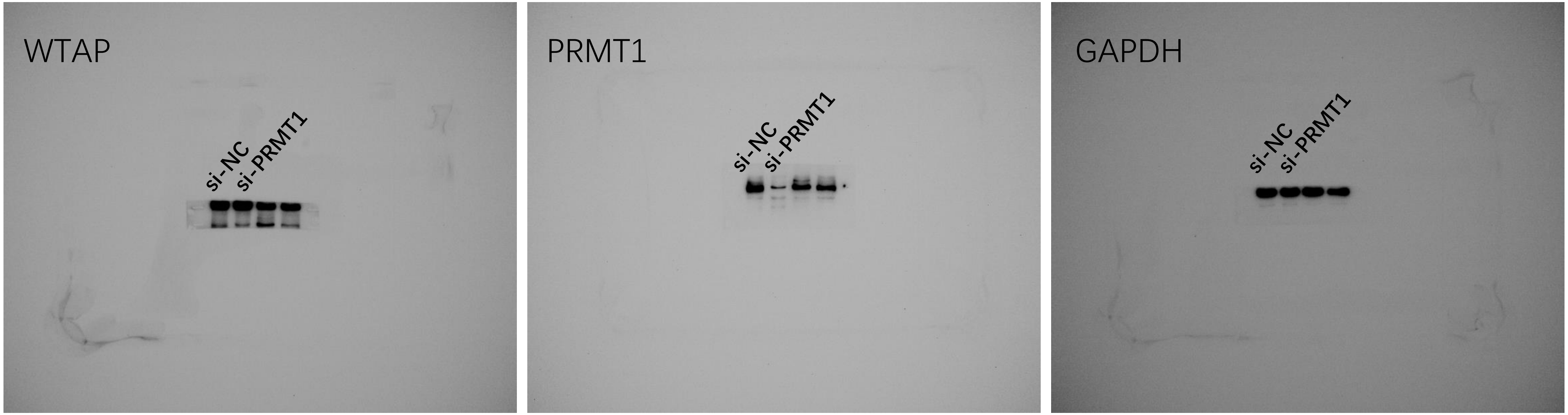

Figure S6

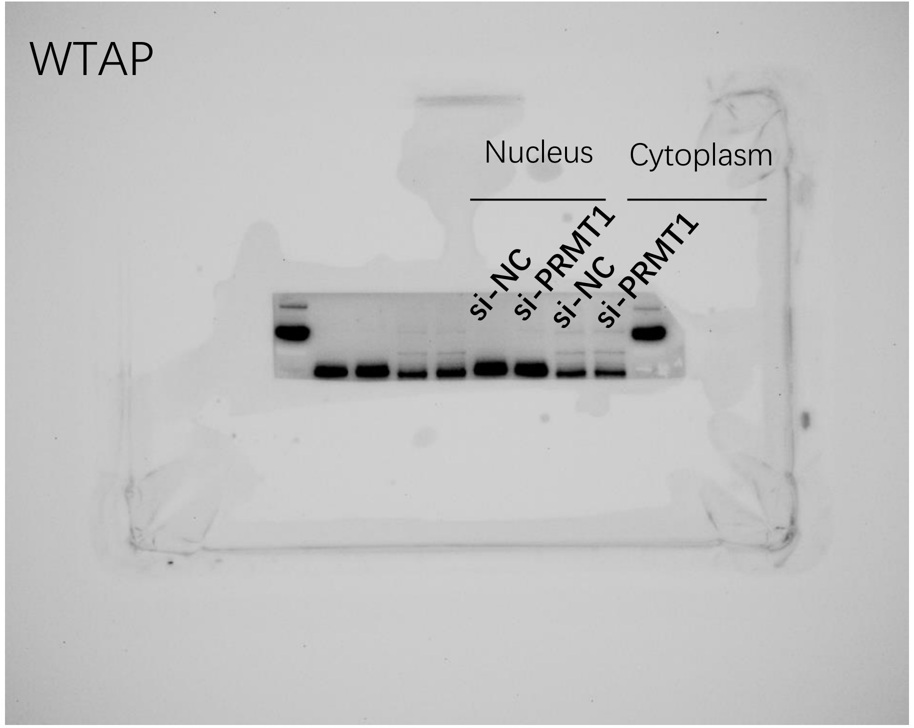
WTAP
Nucleus
Cytoplasm
si-NC
si-PRMT1
si-NC
si-PRMT1

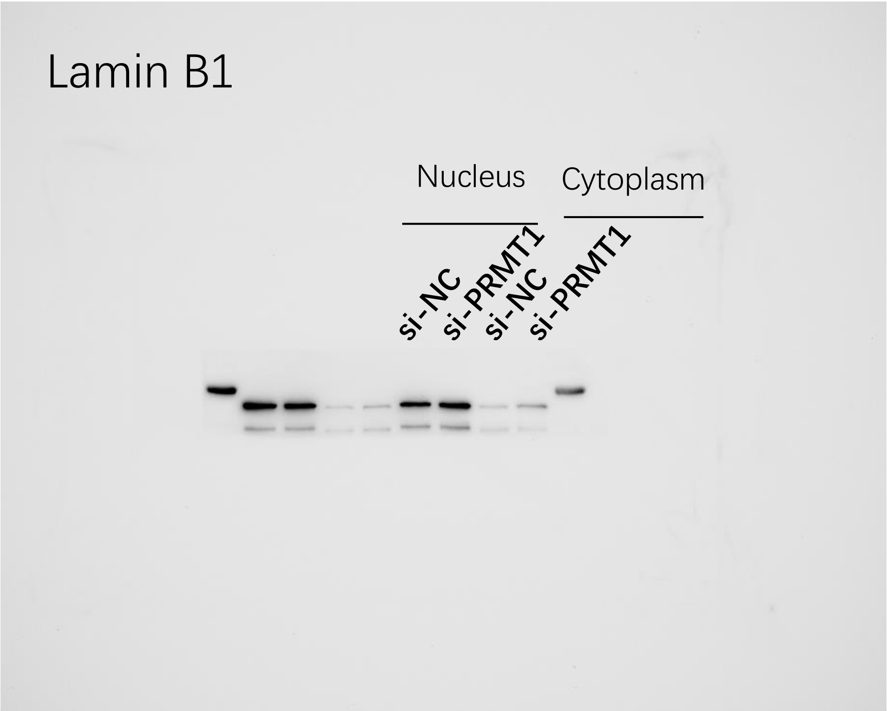
Lamin B1
Nucleus
Cytoplasm
si-NC
si-PRMT1
si-NC
si-PRMT1

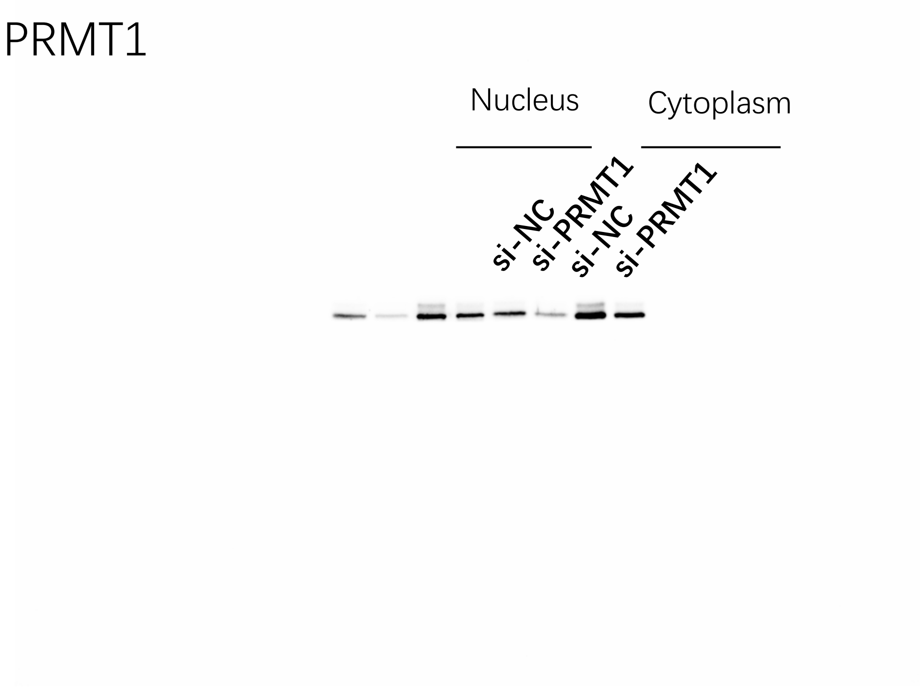
PRMT1
Nucleus
Cytoplasm
si-NC
si-PRMT1
si-NC
si-PRMT1

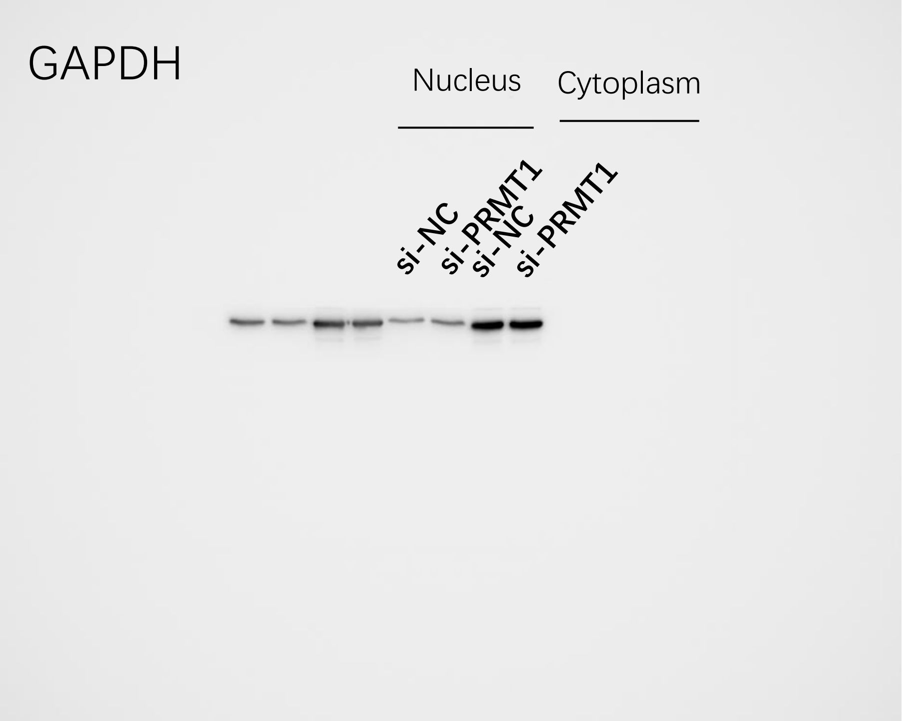
GAPDH
Nucleus
Cytoplasm
si-NC
si-PRMT1
si-NC
si-PRMT1
